# Influence of Different Degrees of Food Restriction on Immune Function and Gut Microbiota in Brandt’s Voles

**DOI:** 10.3390/microorganisms14081630

**Published:** 2026-07-27

**Authors:** Yunqi Liu, Deli Xu

**Affiliations:** School of Life Sciences, Qufu Normal University, Qufu 273165, China; 17562002639@163.com

**Keywords:** Brandt’s voles, food restriction, gut microbiota, immune function

## Abstract

Small mammals in temperate areas frequently face fluctuations in food resources, and food shortage is a key factor affecting their immune function. In order to understand whether immune function and gut microbiota change under food shortage, adult male Brandt’s voles (*Lasiopodomys brandtii*) were used and divided into Fed (Fed, n = 8), 80% food restriction (80% FR, n = 10) and 60% food restriction (60% FR, n = 9) groups. The experimental course lasted for 35 days. Food restriction reduced body mass (*p* < 0.001) but did not affect total fat mass (*p* = 0.532) or blood glucose levels (*p* = 0.777) compared with the Fed voles. Immunological parameters—including wet masses of thymus and spleen, phytohemagglutinin (PHA) responses at 6 h, 12 h, 24 h, and 48 h post PHA injection, interleukin-4 (IL-4) levels (*p* = 0.700), the counts of white blood cells (*p* = 0.864), lymphocytes (*p* = 0.692), and neutrophils (*p* = 0.729)—were not influenced by food restriction, indicating that voles could maintain stable immune function under food shortage. Similarly, leptin (*p* = 0.219) and corticosterone (CORT) (*p* = 0.282) levels were also not impacted by food restriction, and no correlation existed between these two hormones and the above immunological indices. However, interferon-γ (IFN-γ) (*p* < 0.001) levels in the 80% FR group were the highest among the three groups. Food restriction had no effect on alpha (α)-diversity of the gut microbiota, indicating that food restriction does not affect their diversity or richness. Food restriction did not affect relative abundance at the phylum or genus levels, nor at most family levels, but reduced Erysipelotrichaceae at the family level. In summary, Brandt’s voles could maintain stable energy status, leptin and corticosterone levels, and the diversity and richness of the gut microbiota under food restriction, which may help us to understand this species’ ability to survive well in times of food shortage.

## 1. Introduction

The immune system defends animals against the attacks of environmental pathogens including viruses, bacteria and fungi, which is crucial for their survival [1]. Immune function is influenced by numerous environmental factors, such as season [2], photoperiod [3], temperature [4] and food [5]. Food availability is a key factor affecting animal immunity [1]. Generally, food shortage suppresses immune function in animals. For instance, food restriction decreased cellular immune responses in deer mice (*Peromyscus maniculatus*) [6] and Siberian hamsters (*Phodopus sungorus*) [7], or antibody levels in rat-like hamsters (*Cricetulus triton*) [8]. Moreover, food restriction impaired immunological memory and reduced the number of spleen-derived B lymphocytes in deer mice [9,10]. Additionally, the proliferation of T lymphocytes and humoral immunity was suppressed in food restricted mice [11]. However, some investigators have demonstrated that food or caloric restriction has an enhancing effect on immune function [5,12]. Other researchers have found food restriction has no effect on immune function in some species. For example, food restriction did not affect bacterial killing capacity, indicative of innate immunity in Siberian hamsters [5] or cellular and humoral immunity in striped hamsters (*Cricetulus barabensis*) [13] and Mongolian gerbils [1]. It seems that food shortage exerts different effects on distinct components of the immune system across different species. Therefore, further research is needed to illustrate the influences of food shortage on immunity in more species, and the mechanisms by which food restriction affects immune responses deserve further investigation.

Cellular immunity is usually evaluated by phytohemagglutinin (PHA) response in birds and small mammals, which mainly defends against intracellular pathogens [14,15]. Immune organs including thymus and spleen are also indicative of immune function, in which primary T cell development is located in the thymus and a larger spleen indicates stronger immunity [16,17]. In addition, hematological indices such as white blood cells (WBC), lymphocytes (LYMP), intermediate granulocytes (MID), and neutrophilic granulocytes (GRAN) are crucial for fighting against pathogens [18]. Cytokines are proteins or glycoproteins produced by immune cells and play an important role in regulating innate and adaptive immunity [19]. T helper (Th) type 1 cytokines such as interferon-γ (IFN-γ) primarily control intracellular pathogens, and Th1 effector responses are typically dominated by innate and cell-mediated responses [20]. IFN-γ can fight against viral and intracellular bacterial infections and disease in vertebrates [21]. Th2 cytokines, most notably interleukin-4 (IL-4), are mainly responsible for promoting B-cell proliferation, regulating antibody production and inflammation, and hence enhancing antibody-mediated humoral responses and control extracellular pathogens [19].

Leptin is a cytokine-like hormone primarily secreted by white adipose tissue (WAT), and it plays an important regulatory role in energy balance and immune function [22,23]. WAT not only provides energy storage but has also been regarded as an important endocrine and immune organ [24]. Stressors including food shortage often stimulate the hypothalamic/pituitary/adrenal (HPA) axis and subsequently increase the production of stress hormones such as corticosterone (CORT), which usually reduce immune responses [25].

The gut microbiota has developed a symbiotic relationship characterized by mutual dependence and mutual benefit through long-term co-evolution with humans and animals [26]. It plays a crucial role in various physiological processes including immune function, reproduction and metabolism [27,28]. The structure and function of the gut microbiota are influenced by environmental factors such as diet, photoperiod, temperature and season [29]. Food shortage is one of the key factors affecting the gut microbiota. For example, food restriction reduced the abundance-based coverage estimator (ACE) and Chao1 indices in golden snub-nosed monkeys (*Rhinopithecus roxellana*) during spring, indicating a significant decrease in gut microbiota richness [30]. In addition, beta (β)-diversity analysis showed that food restriction affected the community structure of the gut microbiota, and reduced the relative abundances of Firmicutes and Bacteroidota, but enhanced genus Muribaculaceae of the phylum Verrucomicrobiota [30]. Food restriction also increased the Flavobacteriia and Betaproteobacteria classes, the Flavobacteriales and Burkholderiales orders, the Flavobacteriaceae family, and the Un-s-Clostridiaceae genus GM1 in Brandt’s voles (*Lasiopodomys brandtii*) [31]. Additionally, FR increased the abundance of Lactobacillus and Bifidobacterium in mice [32]. However, other studies have found that food restriction has no effect on the α-diversity and β-diversity of the gut microbiota in *Eothenomys miletus* [33]. It appears that food shortage is a critical factor influencing the diversity, abundance and structure of the gut microbiota in animals. As described above, food restriction can affect immune function and the gut microbiota; however, whether changes in the gut microbiota can mediate the effects of food shortage on immune responses remains unclear.

Brandt’s voles (*Lasiopodomys brandtii*) are typical temperate grassland rodents and they are primarily distributed across the Inner Mongolian grasslands of China, Mongolia, and the south-eastern region of Lake Baikal in Russia [30,34]. This species is characterized by gregarious behavior, herbivory and no hibernation. The environmental temperature ranges from –25 °C to –42 °C, and food availability also shows significant seasonal change [35]. Voles feed on plant stems and leaves during spring and summer, and plant seeds in autumn and winter. Therefore, Brandt’s voles often face food shortages in winter, and providing food in winter can substantially improve their winter survival rate, demonstrating that food shortage is an important factor reducing winter survival [36]. However, it remains unclear whether food shortage affects immunity and further survival by altering gut microbiota in this species. Therefore, this study aims to investigate the effects of different degrees of food restriction on the immune function and gut microbiota, and to explore whether changes in gut microbiota mediate the impact of food shortage on immunity in Brandt’s voles. We hypothesized that food restriction would affect immune function and gut microbiota, and we predicted that the suppression of immune function by food restriction might be related to changes in gut microbiota.

## 2. Materials and Methods

### 2.1. Experimental Design

All animal procedures were conducted in accordance with the guidelines of the Biomedical Ethics Committee of Qufu Normal University (Approval number: 2025035). In total, 20 females and 20 males from the indoor breeding population of Brandt’s voles in the Institute of Zoology, Chinese Academy of Sciences, were transported to the animal room of Qufu Normal University in April 2023, and then the experimental breeding colony was formed by paired breeding. The male Brandt’s voles (aged about 3 months) used in this experiment were housed individually in cages (30 cm × 15 cm × 20 cm) with sawdust as the bedding under a 12 h light and 12 h dark photoperiod and the temperature of 23 ± 1 °C. The animals had free access to water. The food (Beijing KeAo Feed Co., Beijing, China) mainly consisted of 6.2% crude fat, 18% crude protein, 23.1% neutral fiber, 5% crude fiber, 12.5% acid detergent fiber and 10.0% ash, and the caloric value was 17.5 kJ/g.

When animals have free access to food, they usually would not die. However, animals may die when faced with food shortages. Therefore, the sample sizes of the food restriction (FR) groups were increased when grouping. Based on the shiny fur color and agile movements of voles, they were considered healthy individuals. According to body masses, 30 male healthy adult male voles were divided into the Fed group (Fed, n = 8), the 80% food restriction group (80% FR, n = 11), and the 60% food restriction group (60% FR, n = 11). The treatment period lasted 35 days. Baseline food intake (g/day) of voles used for food restriction was measured for 9 days (once every 3 days). Specifically, each vole in the food restricted groups was given sufficient food in a quantitative manner, and after 3 days, the remaining food including the leftover or spilled food was collected and weighed to determine the actual daily food intake of each animal. This process was repeated 2 times. The average daily food intake of each vole measured three times was used as the baseline food intake, and the restricted food amount was 80% and 60% of the baseline food intake, respectively. During the experiment, one vole in the 80% FR group died after 20-day food restriction, and two voles in the 60% FR group died after 12 and 14 days of food restriction, respectively. The data of these three voles were not included in the subsequent analysis. Therefore, the sample sizes of the Fed, 80% FR, and 60% FR groups were 8, 10, and 9, respectively. Day 0 and day n represented the initial day and nth day of the experiment, respectively.

### 2.2. Body Composition and Organs

At the end of the experiment, after collecting trunk blood, 20 μL whole blood was diluted immediately in 4 mL diluent for hematological parameters. Other trunk blood samples were placed in ice. After about 1 h, this whole blood was centrifuged at 4 °C at 3500 rpm for 30 min. The supernatant serum was stored at −80 °C for later assays of cytokines and hormones.

Body composition was examined as described previously [15]. In brief, the visceral organs, including heart, thymus, lungs, liver, spleen, kidneys, adrenal glands, testes, epididymis, seminal vesicles and the digestive organs with contents (i.e., stomach, small intestine, caecum and colon), were dissected and weighed (±1 mg). Moreover, subcutaneous fat, retroperitoneal fat, mesenteric fat and perigonadal fat were also dissected carefully and weighed. All the four parts of fat mass were regarded as total body fat mass. Subcutaneous fat content, retroperitoneal fat content, mesenteric fat content, perigonadal fat content and total body fat content were calculated as their respective fat mass divided by final body mass [37].

### 2.3. Cellular Immunity Assay

PHA response indicative of cellular immune response was assessed as described previously [15]. Specifically, the voles on day 32 were caught, then we measured the footpad thickness of each one’s left hind foot with a micrometer (Digimatic Indicator ID-C Mitutoyo Absolute cod. 547-301, Mitutoyo Corporation, Kawasaki, Japan) to ±0.01 mm. Immediately thereafter, voles were injected subcutaneously with 0.1 mg of PHA (PHA-P, Sigma L-8754) dissolved in 0.03 mL of sterile saline in the middle of the footpad. Then, 6 h, 12 h, 24 h and 48 h after injection, we measured footpad thickness. The PHA response (i.e., cellular immunity) was calculated as the difference between pre- and post-injection measurements divided by initial footpad thickness (PHA response = (post PHA–pre PHA)/pre PHA). At each time point (i.e., 0 h, 6 h, 12 h, 24 h, and 48 h post PHA injection), footpad thickness was measured six times to obtain the value of each animal [15]. To minimize errors, all measurements were performed by the same person.

### 2.4. Hematological Parameter Assay

At the end of the experiment, trunk blood was collected and 20 μL whole blood was diluted immediately in 4 mL diluent for hematological parameters. The hematological parameters consisting of white blood cells (WBC), lymphocytes (LYMP), percentage of lymphocytes (LYMP%), neutrophil granulocytes (GRAN), percentage of neutrophil granulocytes (GRAN%), intermediate cells (MID), percentage of intermediate cells (MID%), red blood cells (RBC), hemoglobin concentration (HGB), and platelets (PLT) were counted in the Hematology Analyzer (Auto Counter 910EO+) (SWELAB, Gothenburg, Sweden) [13]. The detailed procedure followed the manufacturer’s instructions of the Hematology Analyzer. Specifically, after powering on the hematology analyzer, the instrument automatically performs pipeline cleaning, pressure detection, and background counting, with the status bar displaying “Ready”. Allow the quality control sample to stand at room temperature for 15 min, then conduct quality control testing for low, medium, and high levels. After passing the tests, thoroughly mix the sample, place it on the test tube rack, and push it into the sample inlet. The system will automatically identify, mix, aspirate, and print the results after testing.

### 2.5. Blood Glucose Assay

Blood glucose level was measured with the Blood Meter (Sinocare, Sanuo GA-3, Changsha, China) according to the manufacturer’s instructions. The range tested of blood glucose was 1.1–33.3. The range tested of blood glucose was 1.1–33.3 mmol/L, and the detailed test steps were described in the previous study [38]. Specifically, the Blood Glucose Test Strip was carefully inserted into the FreeStyle Mini Blood Meter. A drop of blood was dropped onto this Blood Glucose Test Strip in the blood glucose meter when collecting trunk blood at the end of the experiment. After a moment, the value of blood glucose would appear on the display window of the glucose meter [38].

### 2.6. IFN-γ and IL-4 Assays

Serum concentrations of interferon-γ (IFN-γ) and interleukin-4 (IL-4) were determined by hamster IFN-γ (ZY65007-A) and IL-4 (ZY65009-A) ELISA kits (Beijing Boshengsiyuan Biotechnology Co., Ltd., Beijing, China), respectively. The ranges detected by these assays were 100–800 pg/mL and 1–48 pg/mL, respectively, when using a 10 μL sample (see manufacturer’s instructions for hamster IFN-γ and IL-4 RIA Kits). The detailed procedure followed the manufacturer’s instructions of the hamster IFN-γ and IL-4 ELISA kits, respectively. Specifically, the original density standard solution was diluted according to the table in the manufacturer’s instructions. Blank wells without serum samples and HRP-Conjugate reagent were set separately and 50 μL sample dilution was added in each blank well. To each sample well was added 40 μL sample dilution and 10 μL serum, then they were mixed gently. After closing the plate with the closure membrane, the plate was incubated for 30 min at 37 °C. Thereafter, the closure membrane was uncovered, and the liquid in the wells was discarded. The plate was dried by swing, and washing buffer was added to each well. The plate remained still for 30 s and was then drained. The wells were washed 5 times. To each sample well except the blank wells, 50 μL horseradish peroxidase (HRP)-conjugated reagent was added, and then the plate was incubated for 30 min at 37 °C. After washing five times as described above, chromogen solution A (50 μL) and chromogen solution B (50 μL) were added to each well. The plate was protected from light, and was placed at 37 °C for 10 min. Thereafter, 50 μL of stop solution was added to each well and the plate was further incubated for 15 min. The optical density (OD) of each well was determined using a plate reader (Bio-Rad, Benchmark; Richmond, CA, USA) equipped with a 450 nm wavelength filter. The blank well was taken as zero, and the sample wells were read at 450 nm. IFN-γ level was calculated according to the standard curve, which was made according to the known different concentrations of the standard solution. The steps of measuring IL-4 concentration were similar to those of the IFN-γ assay as described above except for the difference in the IL-4 ELISA kit used.

### 2.7. Leptin and Corticosterone Assays

Serum leptin and corticosterone (CORT) concentration were determined by the hamster leptin and CORT ELISA kits (numbers of leptin and CORT were ZY5998-A and ZY6006-A, Beijing Boshengsiyuan Biotechnology Co., Ltd., Beijing, China). The range of leptin and CORT detected by these assays was 0.3–8 ng/mL and 5–240 ng/mL, respectively, when using a 10 μL sample (see manufacturer’s instructions for hamster leptin and CORT ELISA Kits, respectively). The detailed procedures followed the manufacturer’s instructions for the hamster leptin and CORT ELISA kits, respectively. Specifically, the original density standard solution was diluted according to the table in the manufacturer’s instructions. Blank wells without serum samples and HRP-Conjugate reagent were set separately and 50 μL sample dilution was added in each blank well. To each sample well was added 40 μL sample dilution and 10 μL serum, then they were mixed gently. After closing the plate with the closure membrane, the plate was incubated for 30 min at 37 °C. Thereafter, the closure membrane was uncovered, and the liquid in the wells was discarded. The plate was dried by swing, and washing buffer was added to each well. The plate remained still for 30 s and was then drained. The wells were washed 5 times. To each sample well except the blank wells was added 50 μL horseradish peroxidase (HRP)-conjugated reagent was added, and then the plate was incubated for 30 min at 37 °C. After washing five times as described above, chromogen solution A (50 μL) and chromogen solution B (50 μL) were added to each well. The plate was protected from light, and was placed at 37 °C for 10 min. Thereafter, 50 μL of stop solution was added to each well and the plate was further incubated for 15 min. The optical density (OD) of each well was determined using a plate reader (Bio-Rad, Benchmark, Richmond, CA, USA) equipped with a 450 nm wavelength filter. The blank well was taken as zero, and the sample wells were read at 450 nm. Leptin level was calculated according to the standard curve, which was made according to the known different concentrations of the standard solution [38]. The steps of measuring CORT concentration were similar to those of the leptin assay as described above except for the difference in the CORT ELISA kit used.

### 2.8. Gut Microbiome Analysis

Genomic DNA was extracted from the cecal contents of Brandt’s voles using the cetyltrimethylammonium bromide (CTAB) method. The concentration and purity of the extracted DNA were assessed by electrophoresis on 1% agarose gel. Based on DNA concentration, samples were diluted with sterile deionized water to a final concentration of 1 ng/μL. The V3–V4 region of the 16S rRNA gene was amplified using the universal primer set 341F and 806R with sample-specific barcodes.

PCR products were purified using magnetic bead purification and then mixed with an equal volume of 1 × TAE buffer for electrophoretic detection on 2% agarose gel. Sequencing libraries were constructed using the NEBNext^®^ Ultra™ II DNA (New England Biolabs, Ipswich, MA, USA) Library Prep Kit, and library quality was assessed using Qubit fluorometry and quantitative PCR (Q-PCR). Qualified libraries were subsequently sequenced on the Illumina NovaSeq platform (Manufactured by Illumina, Inc., San Diego, CA, USA) using paired-end sequencing.

### 2.9. Statistical Analysis

Statistical analysis was performed using SPSS 27.0. Prior to statistical analysis, all data were subjected to Kolmogorov–Smirnov and Levene tests to assess normality and homogeneity of variance respectively. All parameters were normally distributed. Body mass, changes in body mass, and changes in PHA response with time were analyzed by using Repeated Measures variance of General Linear Model (GLM). The differences among the Fed, 80% FR and 60% FR groups at a certain time point were analyzed by one-way ANOVA followed by Tukey’s post hoc tests.

Group differences in organ masses with body mass as the covariate were analyzed by GLM multivariate analysis followed by Bonferroni post hoc tests. Group differences in other parameters (body compositions, hematological parameters, PHA response, blood glucose, IFN-γ, IL-4, leptin and corticosterone) were analyzed by one-way ANOVA followed by Tukey’s post hoc tests. Pearson correlation analysis was performed to determine the correlations of immunological parameters including thymus, spleen, hematological parameters, PHA response with body energy status (body fat and blood glucose), leptin, CORT and gut microbiota. The results are presented as the mean ± SE, and *p* < 0.05 was considered to be statistically significant.

## 3. Results

### 3.1. Body Mass

**Figure 1:**
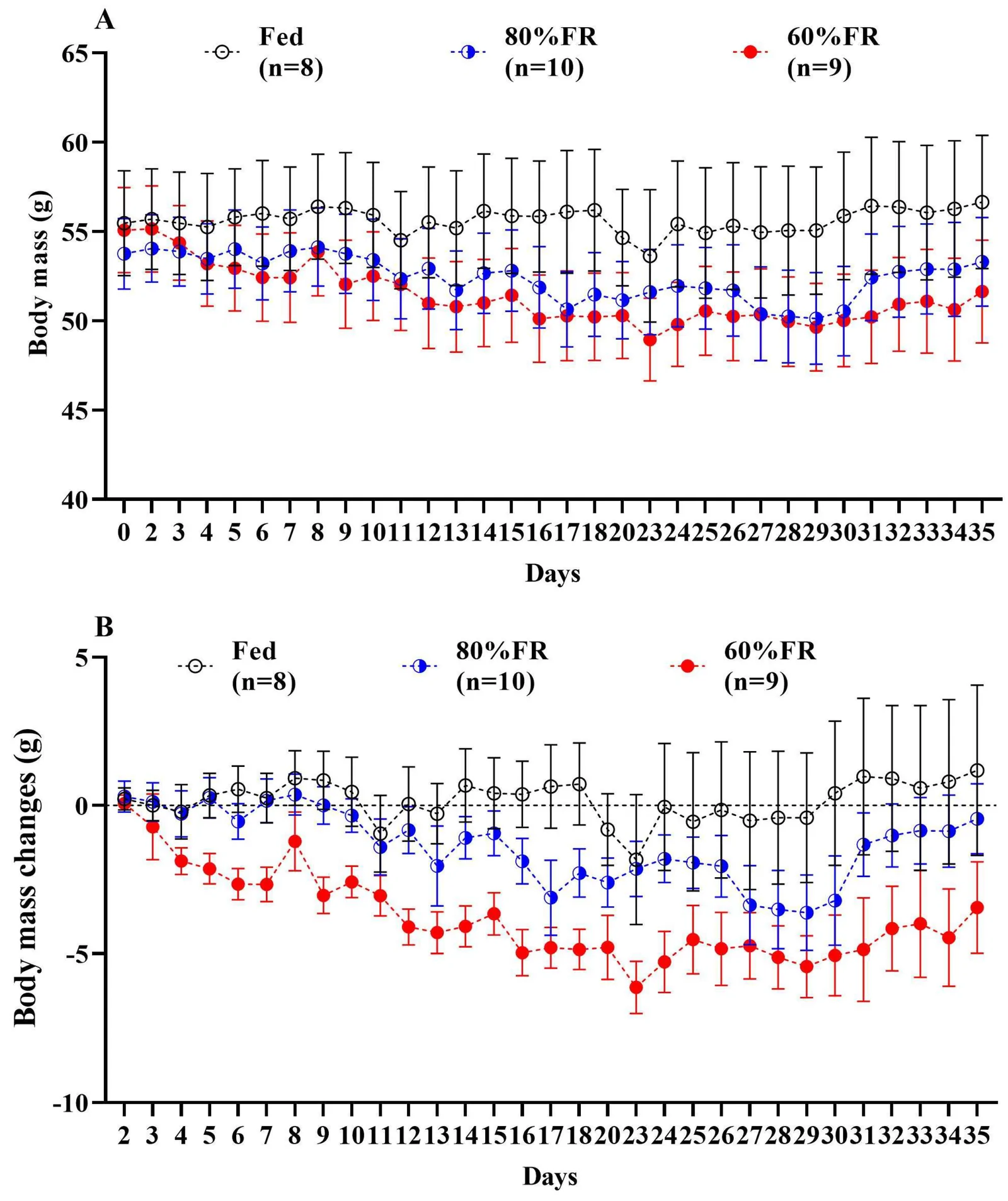
Effect of food restriction on body mass (**A**) and the changes in body mass (**B**) in Brandt’s voles. *p* values of the treatment time, groups and the interaction of treatment time × groups in (**A**) were <0.001, 0.510 and 0.196, respectively. *p* values of the treatment time, groups and the interaction of treatment time × groups in (**B**) were <0.001, 0.031 and 0.434, respectively. FR indicates food restriction.

At the beginning of the experiment, there were no significant differences in body mass among the Fed, 80% FR and 60% FR groups (*p* = 0.918) (Figure 1A). Body mass varied significantly during the course of the experiment (*p* < 0.001). Concretely, body mass in the Fed group did not change over time (*p* = 0.998), while body mass in the 80% FR (*p* < 0.001) and 60% FR (*p* < 0.001) groups decreased significantly with time. Body mass was not affected by groups (*p* = 0.510) or the interaction of treatment time × groups (*p* = 0.196). No significant differences were observed at any time from Day 2 (*p* = 0.879) to Day 35 (*p* = 0.515) (Figure 1A).

Changes in body mass varied significantly with treatment time (*p* < 0.001), and there were significant differences among the three groups (*p* = 0.031), but the interaction of treatment time × group was not significant (*p* = 0.434) (Figure 1B). From Day 5 (*p* = 0.020) to Day 21 (*p* = 0.038), the changes in body mass in the 80% FR and 60% FR groups were all significantly lower than those in the Fed group (Figure 1B).

### 3.2. Body Composition

Food restriction did not affect wet carcass mass, subcutaneous, mesenteric, retroperitoneal, perigonadal and total body fat masses and their respective percentage contents in Brandt’s voles (Table 1). Compared with the Fed group, total body fat masses in the 80% FR and 60% FR groups were reduced by 7.7% and 23.7%, respectively.

### 3.3. Organs

Brain wet mass was the lightest in the 80% FR group among the three groups. The masses of other organs such as thymus, heart, lungs, testes, kidneys and the digestive organs with contents (i.e., stomach, small intestine, caecum and colon) were not affected by food restriction (Table 2).

### 3.4. PHA Response

**Figure 2:**
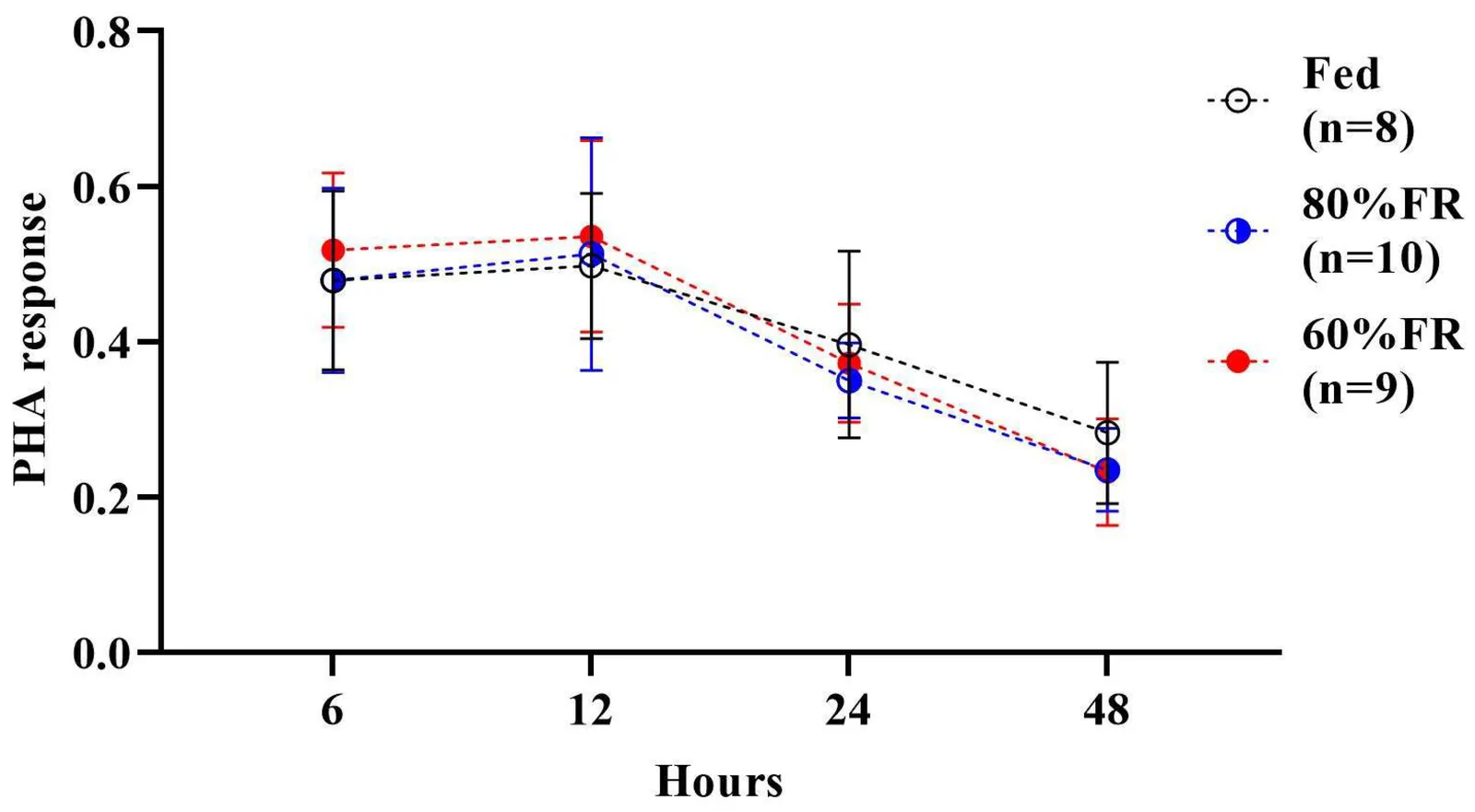
Effect of food restriction on the PHA response in Brandt’s voles. *p* values of the treatment time, groups and the interaction of treatment time × groups were <0.001, 0.832 and 0.517, respectively. SEMs of the Fed groups at 6 h, 12 h, 24 h, 48 h were 0.040, 0.045, 0.029 and 0.025, respectively. SEMs of the 80% FR group at 6 h, 12 h, 24 h, 48 h were 0.035, 0.040, 0.026 and 0.022, respectively. SEMs of the 60% FR group at 6 h, 12 h, 24 h, 48 h were 0.037, 0.042, 0.028 and 0.024, respectively. FR indicates food restriction, and PHA indicates phytohemagglutinin.

PHA responses at 6 h, 12 h, 24 h and 48 h stand for PHA responses 6 h, 12 h, 24 h and 48 h after injection of PHA solution, respectively. PHA response decreased gradually with time (*p* < 0.001), and the maximum value occurred after 12 h injection of PHA solution. Food restriction had no effect on PHA response at 6 h (*p* = 0.714), 12 h (*p* = 0.817), 24 h (*p* = 0.549) or 48 h (*p* = 0.289). The interaction of treatment time × groups was not significant (6 h: *p* = 0.714; 12 h: *p* = 0.817; 24 h: *p* = 0.549; 48 h: *p* = 0.289) (Figure 2). No correlations were observed between total body fat mass and PHA response at 6 h, 12 h, 24 h, and 48 h (Table 3).

### 3.5. Hematological Parameters

Food restriction had no significant effect on WBC, LYMP, GRAN, MID, RBC, HGB, PLT and their percentage in Brandt’s voles (Table 4). The correlation was not observed between WBC, LYMP, GRAN, MID, RBC, HGB, PLT and total body fat mass (Table 3).

### 3.6. Blood Glucose

**Figure 3:**
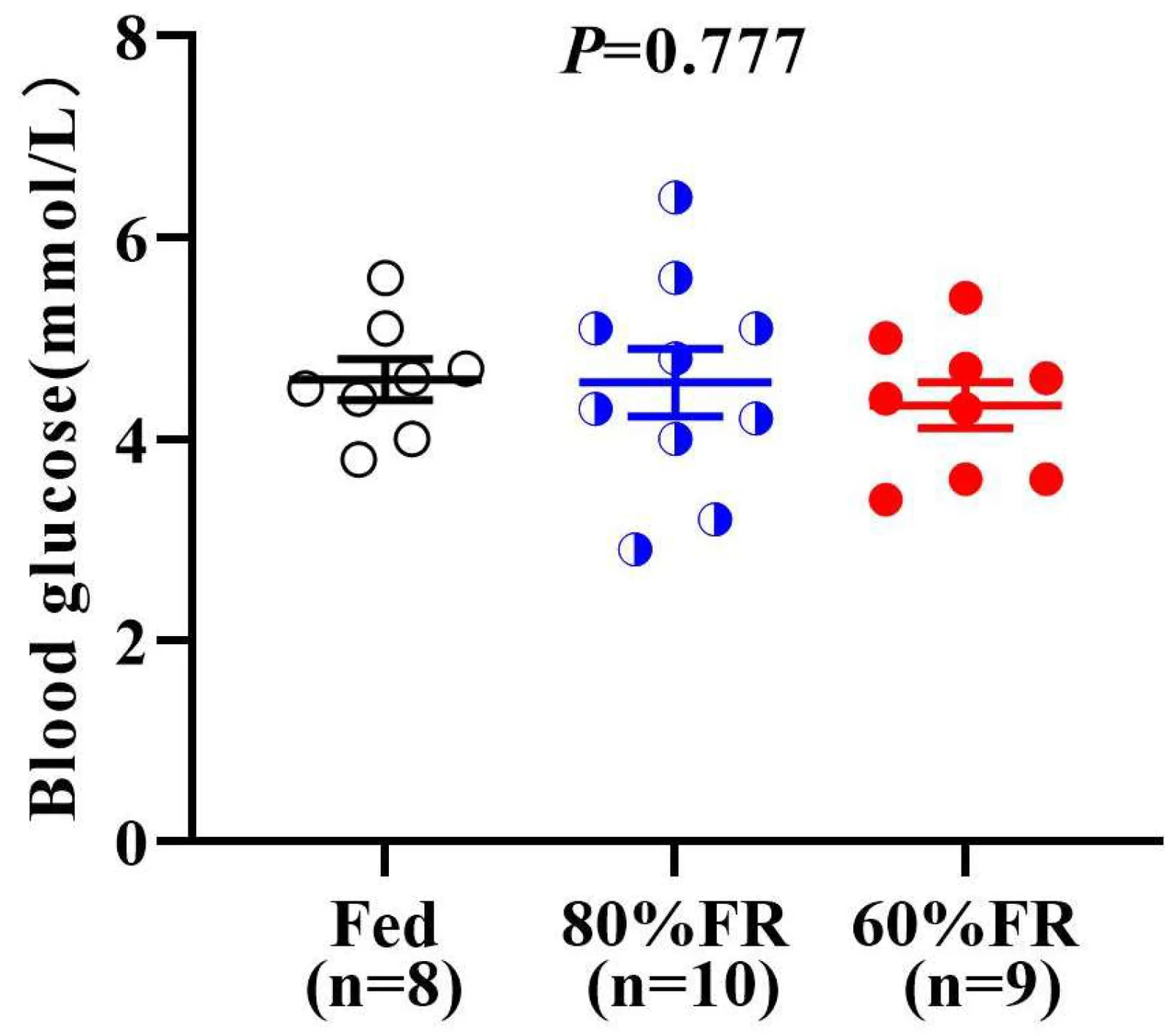
Effect of food restriction on the blood glucose in Brandt’s voles. SEMs of the Fed, 80% FR and 60% FR groups were 0.291, 0.260 and 0.274, respectively. FR indicates food restriction.

Food restriction had no significant effect on blood glucose levels (*p* = 0.777) (Figure 3). There is a positive correlation between blood glucose level and PHA response at 12 h; however, there is no correlation between blood glucose level and total body fat mass or hematological parameters (Table 3).

### 3.7. IFN-γ and IL-4

**Figure 4:**
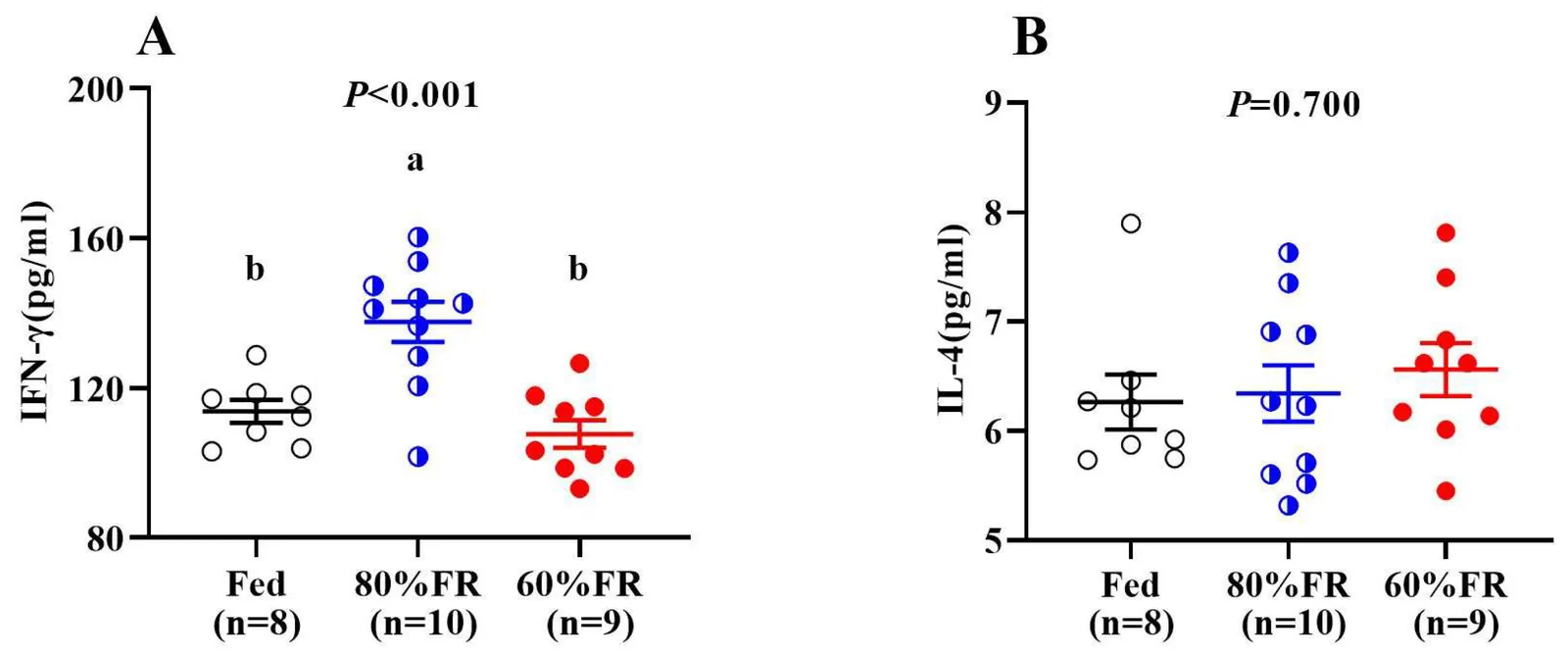
Effect of food restriction on the levels of IFN-γ (**A**) and IL-4 (**B**) in Brandt’s voles. Different letters (i.e., a or b) above scattered dots indicate significant differences between the groups. SEMs of the Fed, 80% FR and 60% FR groups in (**A**) were 5.323, 4.694 and 6.298, respectively. SEMs of the Fed, 80% FR and 60% FR groups in (**B**) were 0.277, 0.244 and 0.328, respectively. FR indicates food restriction. Note: IFN-γ: interferon-γ; IL-4: interleukin-4.

IFN-γ levels in the 80% FR group were the highest among the three groups (*p* < 0.001). However, IL-4 levels did not differ among the three groups (*p* = 0.700) (Figure 4). The levels of IFN-γ and IL-4 were not correlated with total body fat mass, blood glucose or PHA response at 12 h, 24 h, and 48 h (Table 3).

### 3.8. Leptin and CORT

**Figure 5:**
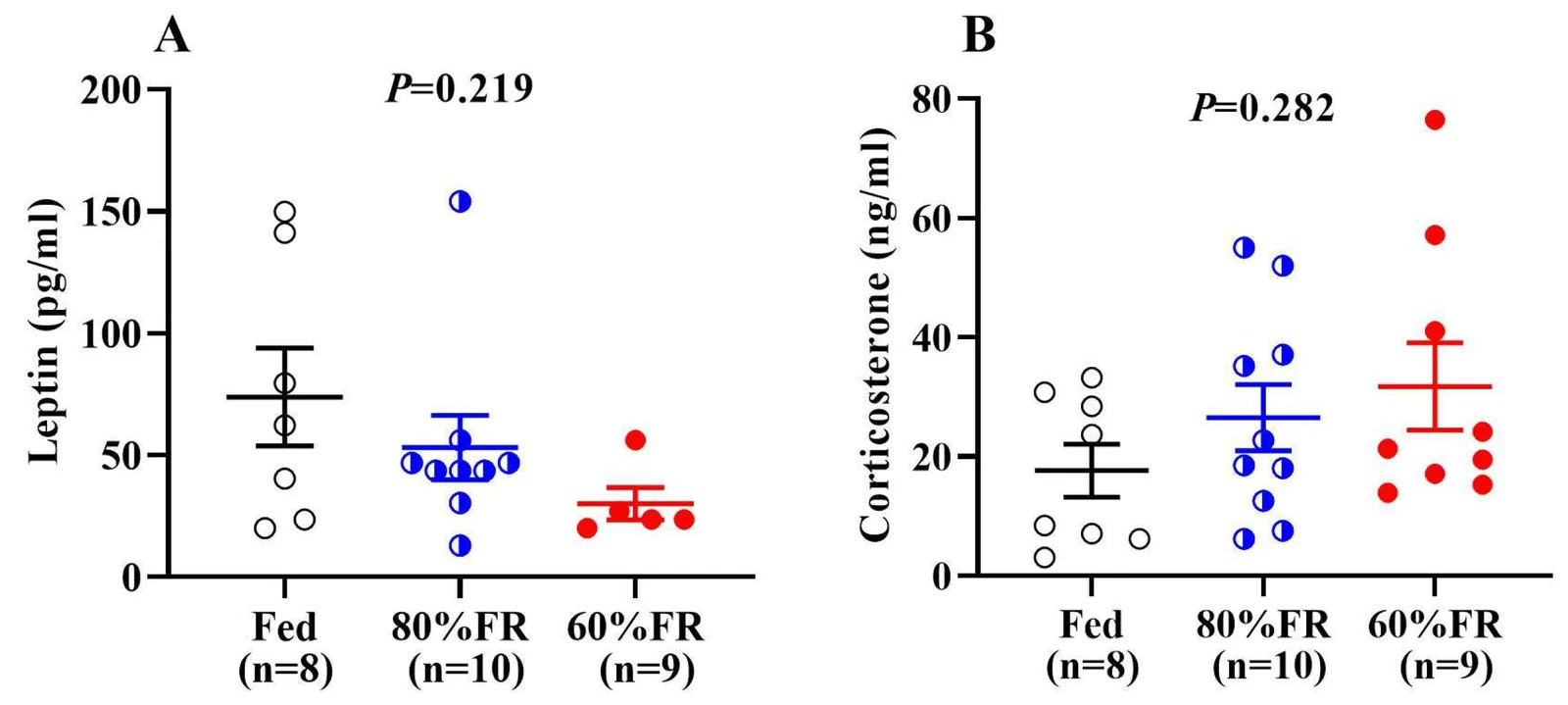
Effect of food restriction on the levels of leptin (**A**) and corticosterone (**B**) in Brandt’s voles. SEMs of the Fed, 80% FR and 60% FR groups in (**A**) were 15.563, 13.726 and 18.415, respectively. SEMs of the Fed, 80% FR and 60% FR groups in (**B**) were 6.651, 5.865 and 7.869, respectively. FR indicates food restriction.

Food restriction had no influence on the levels of leptin (*p* = 0.219) and CORT (*p* = 0.282) (Figure 5). The levels of leptin and CORT were not correlated with total body fat mass, blood glucose, or PHA response at 6 h, 12 h, 24 h, and 48 h (Table 3).

### 3.9. Gut Microbiota Composition

**Figure 6:**
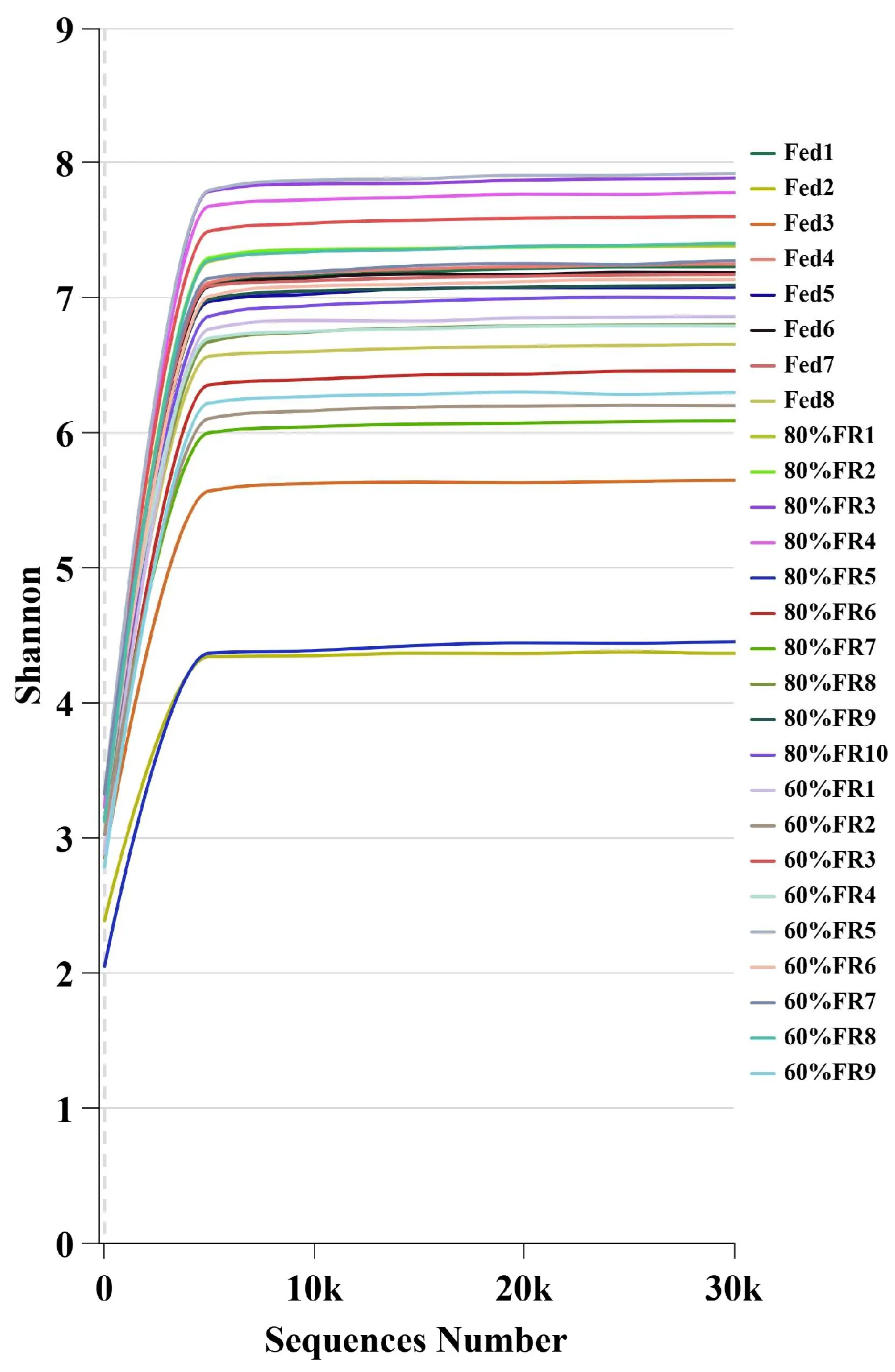
Rarefaction curves of gut microbiota under different degrees of food restriction in Brandt’s voles.

**Figure 7:**
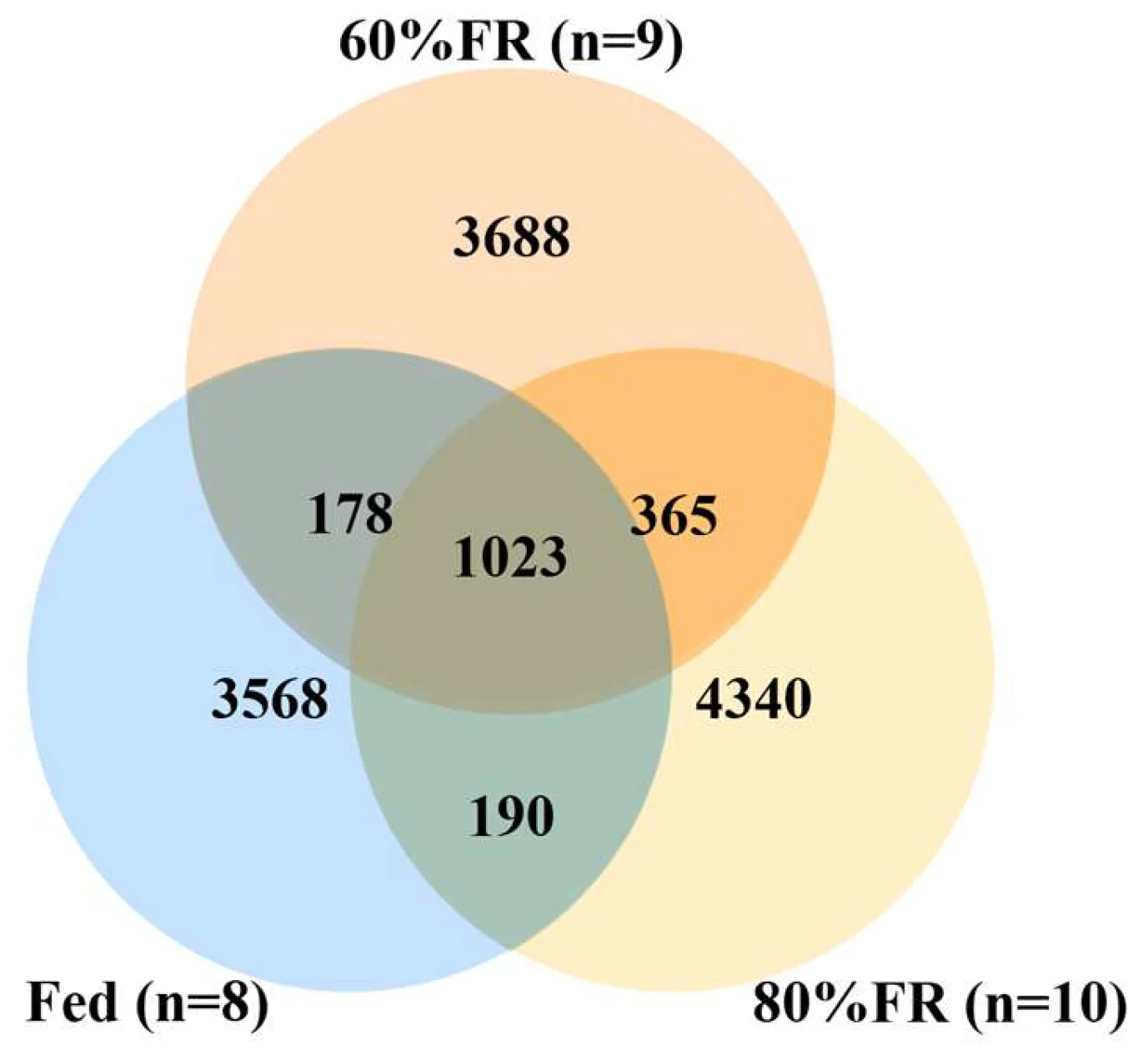
Venn diagram of ASV sequences of gut microbiota under different degrees of food restriction in Brandt’s voles. FR indicates food restriction.

A total of 15,147 ASV sequences were obtained from 27 cecal samples of Brandt’s voles. Compared with the database, all samples were annotated to 18 phyla, 26 classes, 63 orders, 88 families, 154 genera and 48 species. The dilution curve shows that the curve tends to flatten as the extraction number increases (Figure 6), indicating that the sequencing data volume is gradually becoming reasonable and can reflect information on the vast majority of microorganisms in the sample. The findings showed that 1023 ASVs were common to the Fed, 80% FR and 60% FR groups, 3568 ASVs were unique to the Fed group, 4340 ASVs were unique to the 80% FR group, and 3688 ASVs were unique to the 60% FR group (Figure 7).

The Firmicutes and Bacteroidota phyla were the dominant phyla across the three groups, and their relative abundances were 55.5% and 32.5%, respectively. Phyla such as Desulfobacterota and Spirochaetota were also dominant and accounted for 5.8% and 4.2%, respectively. Food restriction had no influence on the relative abundances of the gut microbiota at the phylum level in Brandt’s voles (Figure 7).

**Figure 8:**
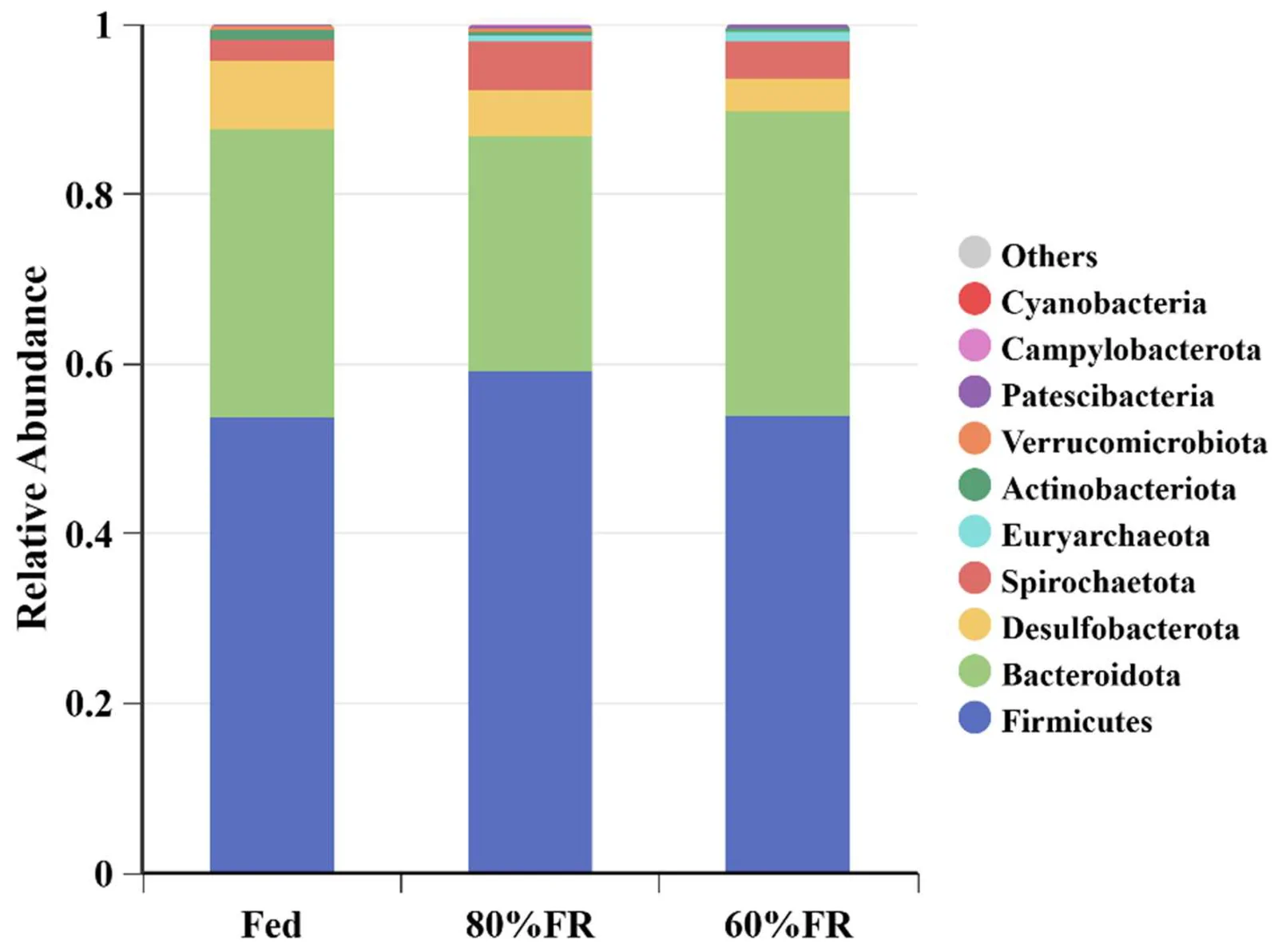
Relative abundance of gut microbiota at the phylum level in Brandt’s voles. FR indicates food restriction.

**Figure 9:**
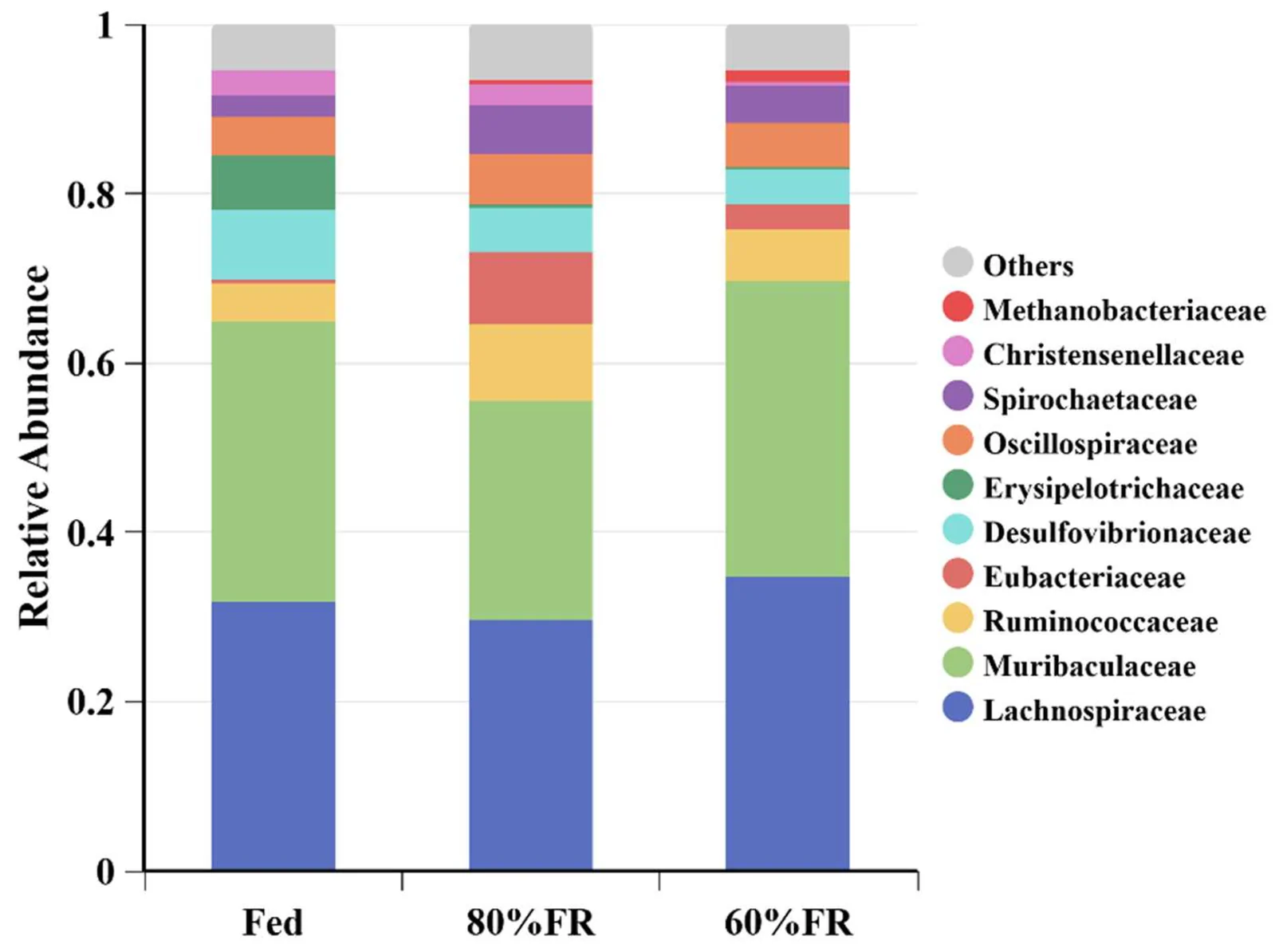
Relative abundance of gut microbiota at the family level in Brandt’s voles. FR indicates food restriction.

At the family levels, the dominant families of the gut microbiota were Muribaculaceae and Lachnospiraceae, their relative abundances being 31.2% and 32.0%, respectively. The relative abundances of other families including Eubacteriaceae, Erysipelotrichaceae, Desulfovibrionaceae, Ruminococcaceae, Christensenellaceae, Spirochaetaceae and Oscillospiraceae were 3.9%, 2.4%, 5.8%, 6.7%, 1.9%, 4.2% and 5.3%, respectively (Figure 8). Food restriction reduced the abundance of the Erysipelotrichaceae family compared with the Fed group (*p* = 0.047) (Figure 9).

**Figure 10:**
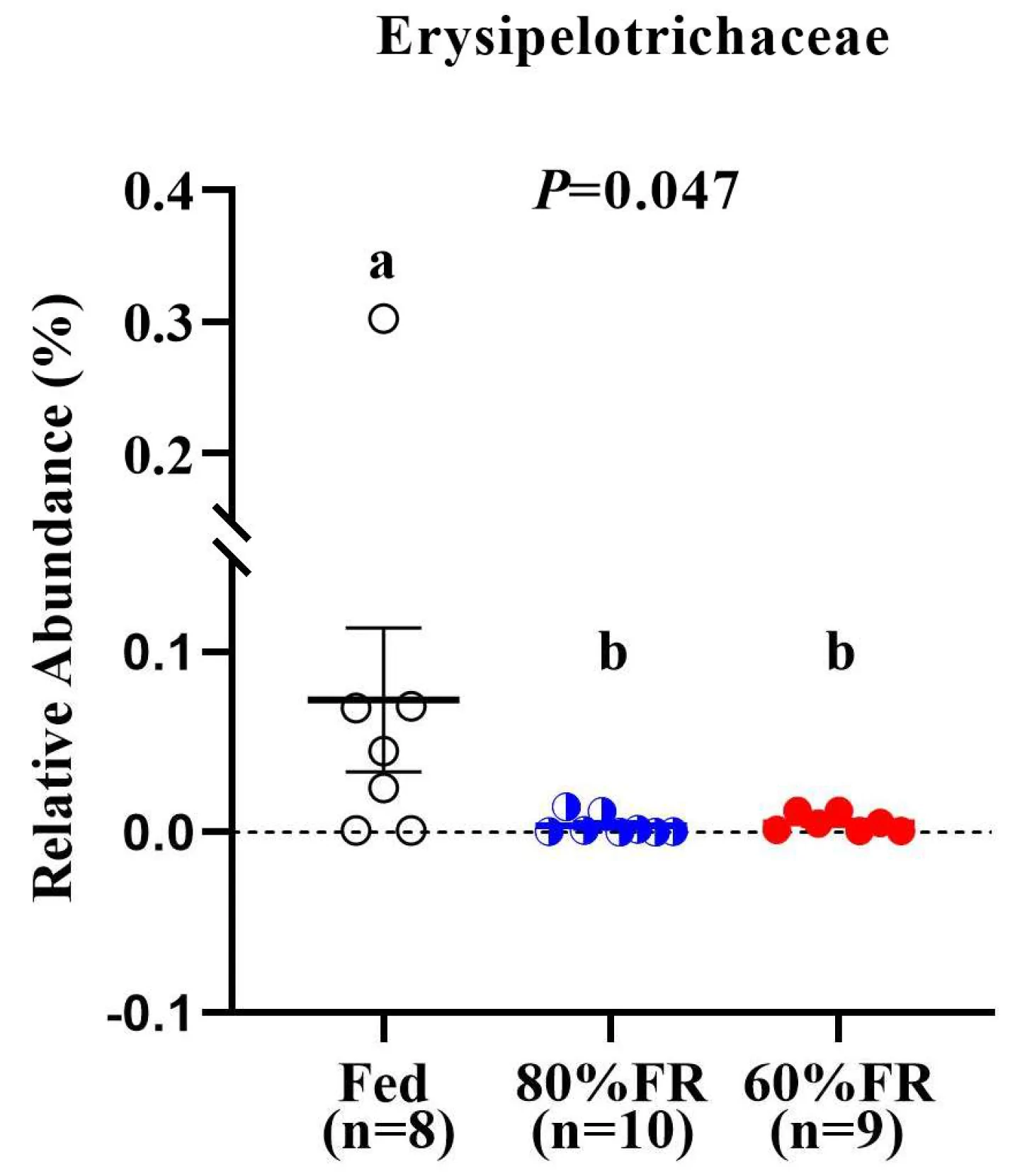
Relative abundance of gut microbiota of Erysipelotrichaceae in Brandt’s voles. SEMs of the Fed, 80% FR and 60% FR groups were 0.019, 0.017 and 0.018, respectively. FR indicates food restriction. Different letters (i.e., a or b) above scattered dots indicate significant differences between the groups.

The dominant genera were *unidentified_Lachnospiraceae* (1.56%), *Desulfovibrio* (5.78%), *unidentified_Ruminococcaceae* (1.04%), *Lachnospiraceae_NK4A136_group* (8.35%), *[Eubacterium]_siraeum_group* (2.09%), *Ruminococcus* (2.12%), and *[Eubacterium]_xylanophilum_group* (1.92%). However, the relative abundances of the remaining genera were relatively low. Food restriction decreased the relative abundance of *unidentified_Lachnospiraceae* but increased the relative abundances of *[Eubacterium]_siraeum_group* and *Methanobrevibacter* (Figure 10).

### 3.10. Alpha and Beta Diversities of Gut Microbiota

**Figure 11:**
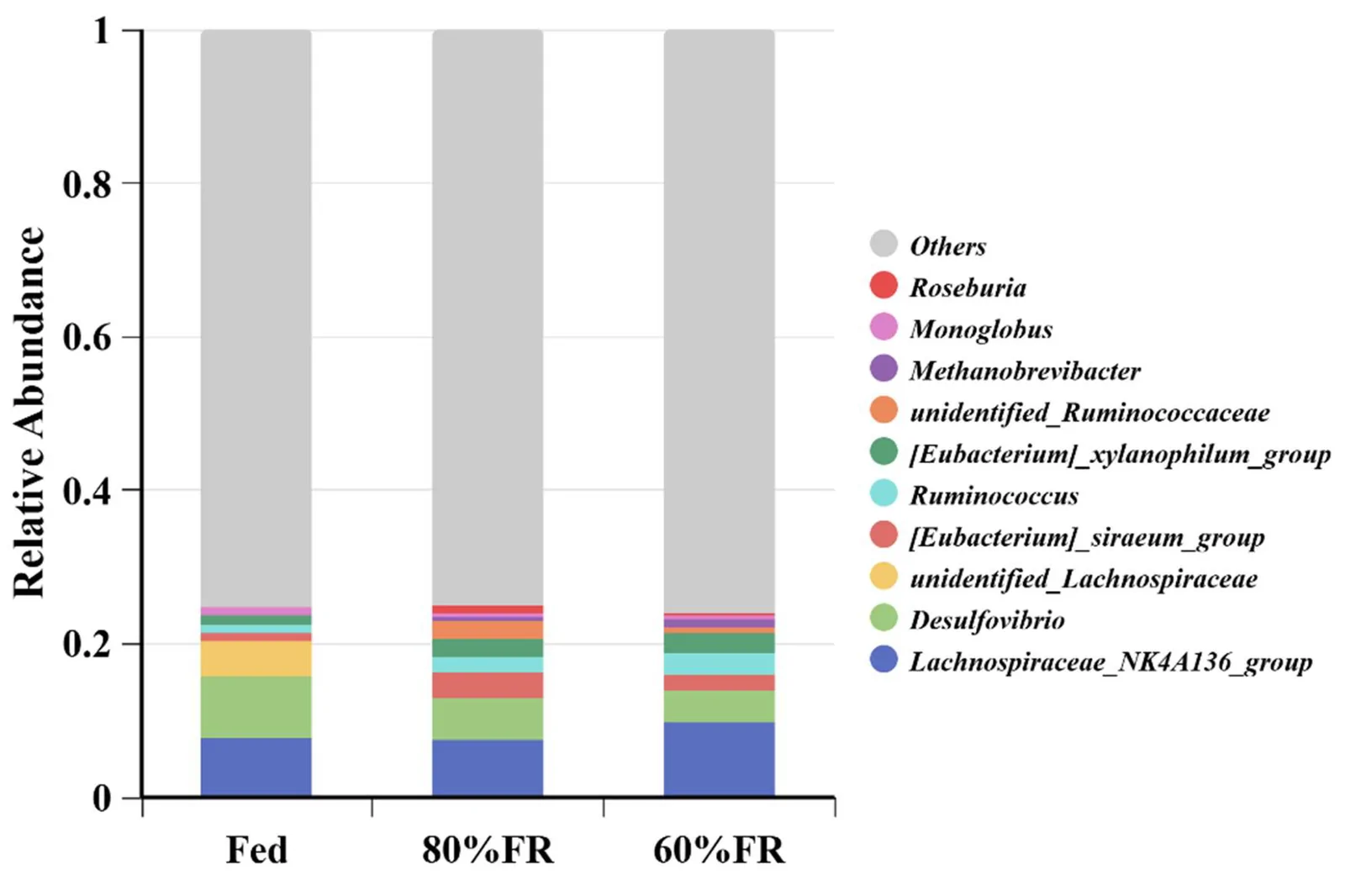
Relative abundance of gut microbiota at the genus level in Brandt’s voles. FR indicates food restriction.

Food restriction had no significant effect on the indices of Chao1 (*p* = 0.710), Simpson (*p* = 0.581), Pielou evenness (Pielou_e) (*p* = 0.541) and Shannon (*p* = 0.557), implying food restriction did not affect α-diversity in Brandt’s voles (Figure 11).

**Figure 12:**
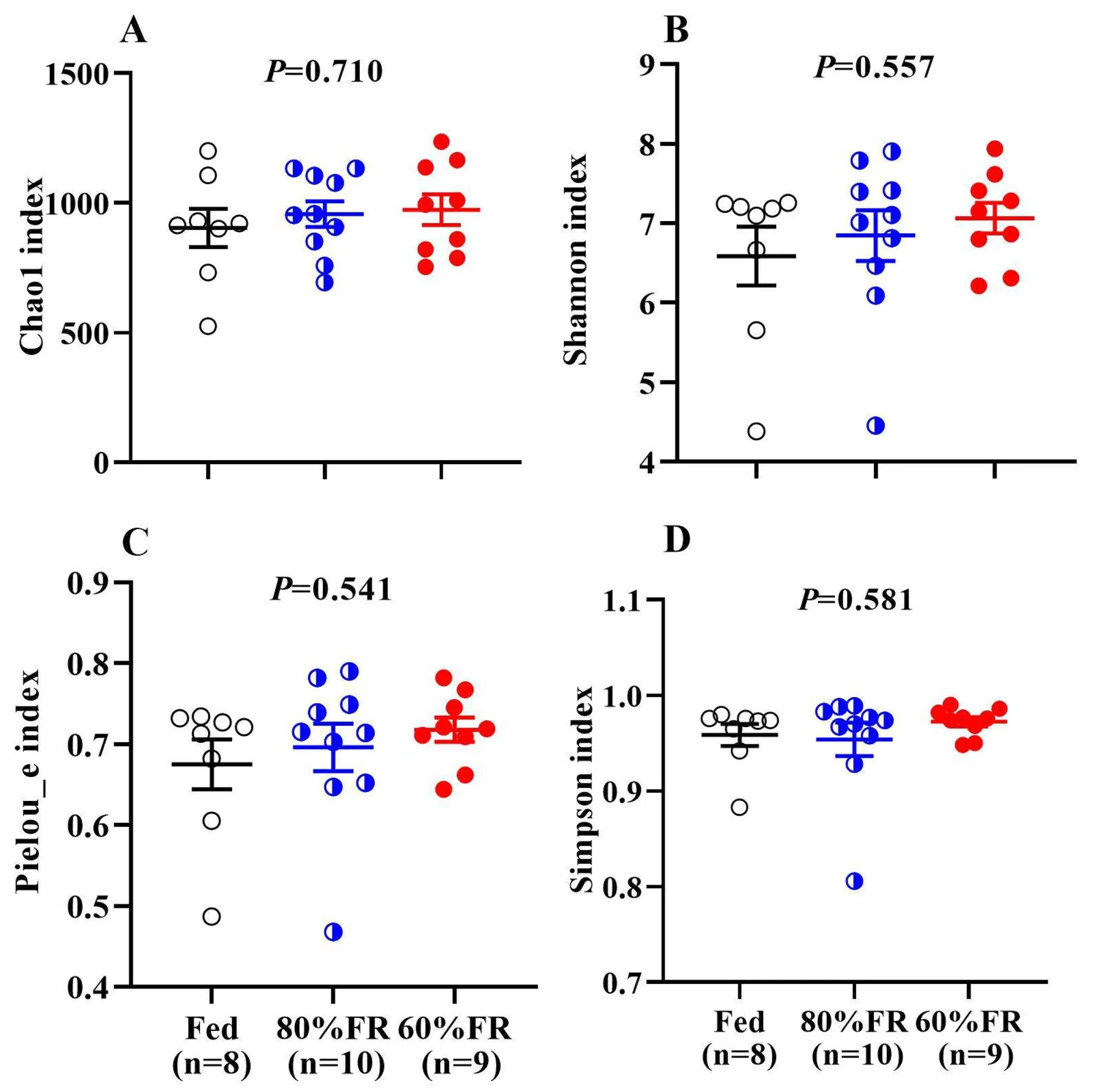
Effect of different degrees of food restriction on the α-diversity Chao1 (**A**), Shannon (**B**), Pielou_e (**C**), Simpson (**D**) of gut microbiota in Brandt’s voles. SEMs of the Fed, 80% FR and 60% FR groups in (**A**) were 63.624, 56.907 and 59.985, respectively. SEMs of the Fed, 80% FR and 60% FR groups in (**B**) were 0.014, 0.012 and 0.013, respectively. SEMs of the Fed, 80% FR and 60% FR groups in (**C**) were 0.028, 0.025 and 0.026, respectively. SEMs of the Fed, 80% FR and 60% FR groups in (**D**) were 0.318, 0.284 and 0.300, respectively. FR indicates food restriction.

The PCoA analysis based on weighted Unifrac distances demonstrated that there were no differences in the structure and composition of the gut microbiota among the Fed and the FR groups (Fed vs. 60% FR: *p* = 0.523; Fed vs. 80% FR: *p* = 0.344; 80% FR vs. 60% FR: *p* = 0.764). However the unweighted Unifrac distances showed that there were differences in the structure and composition of the gut microbiota among the Fed and the FR groups (Fed vs. 60% FR: *p* = 0.011; Fed vs. 80% FR: *p* = 0.586; 80% FR vs. 60% FR: *p* = 0.022), indicating that food restriction has effects on the beta diversity of the gut microbiota (Figure 12).

### 3.11. LEfSe Analysis and Functional Prediction of Gut Microbiome

**Figure 13:**
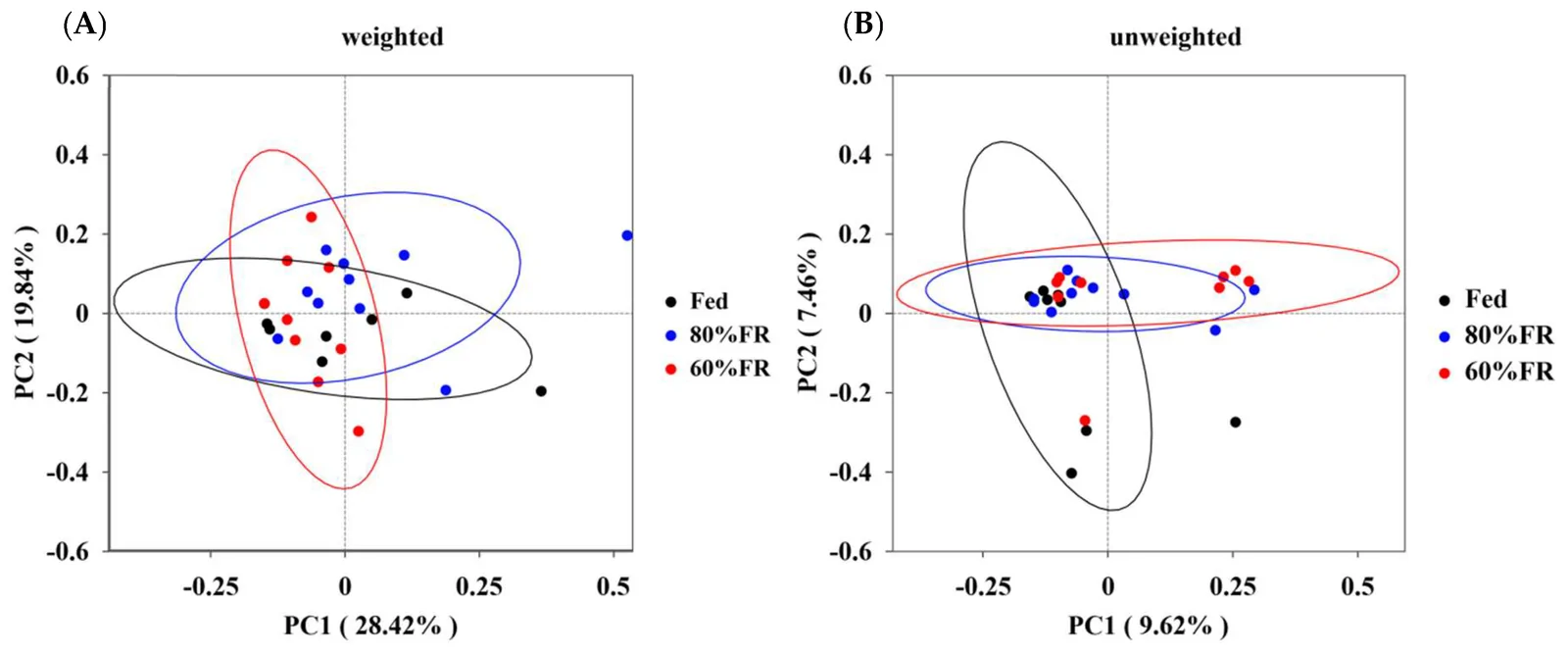
Two-dimensional PCoA plot of gut microbiota of β-diversity in Brandt’s voles. (**A**) Based on weighted Unifrac distance. (**B**) Based on unweighted Unifrac distance. FR indicates food restriction.

LEfSe (LDA Effect Size) analysis indicated that the order Eubacteriales and the family Eubacteriaceae in the 80% FR group differed from those of the Fed and 60% FR groups (Figure 13).

**Figure 14:**
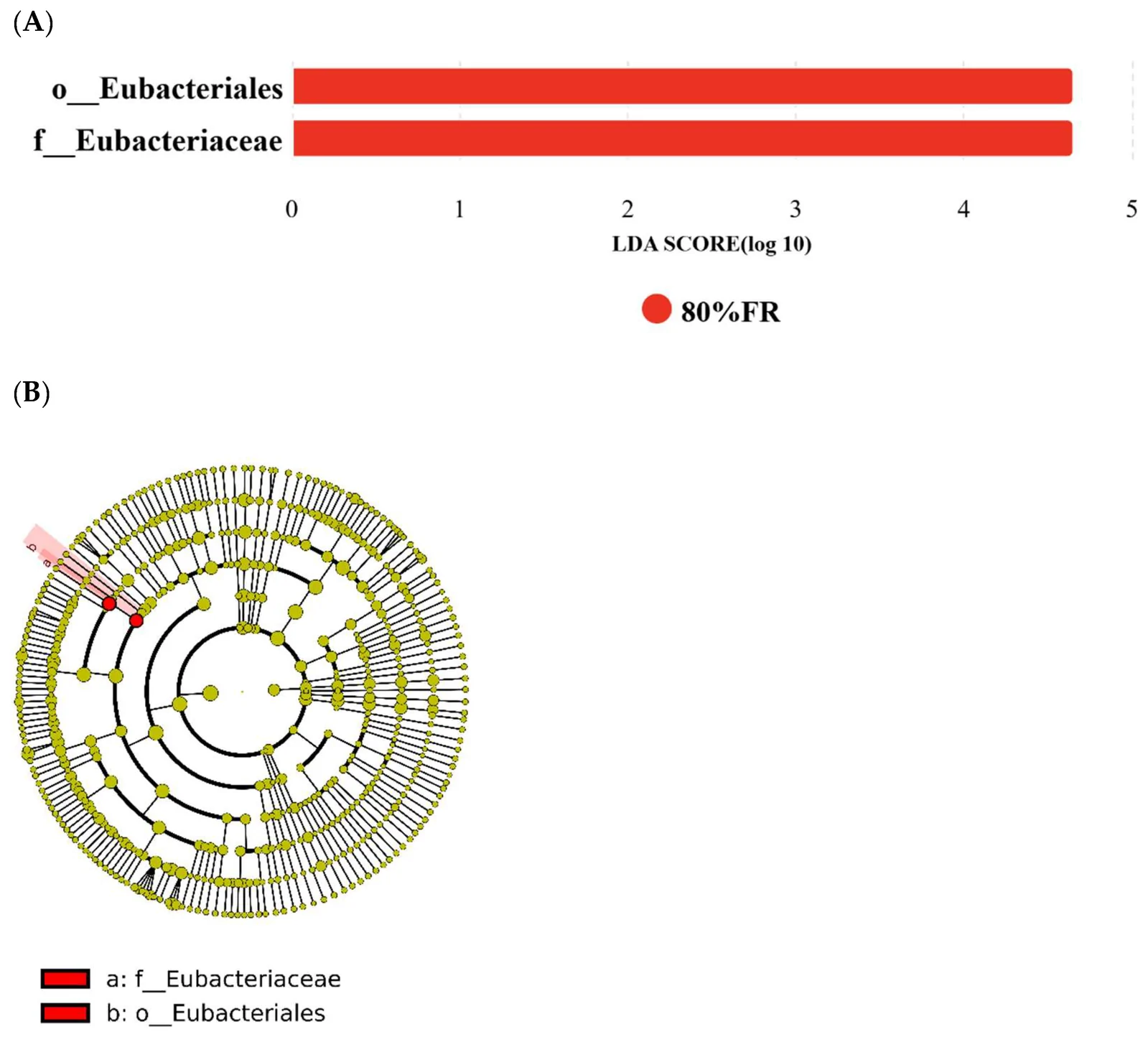
LEfSe analyses of the gut microbiota of the three treatment groups. (**A**) Bar chart showing the distribution of LDA values. (**B**) Phylogenetic tree. FR indicates food restriction. LDA indicates linear discriminant analysis.

Function predictive analyses showed that food restriction had an effect on the function of the gut microbiota at levels 1 and 3. At level 1, 80% FR upregulated environmental information processing, cellular processes and organismal systems, and 60% FR enhanced metabolic function. At level 3, food restriction increased the functional pathways such as transporters, carbon fixation pathways in prokaryotes, glycine, serine and threonine metabolism, bacterial motility proteins, two-component systems, and butanoate metabolism (Figure 14).

### 3.12. Correlations Between Immunological Parameters and Gut Microbiota

**Figure 15:**
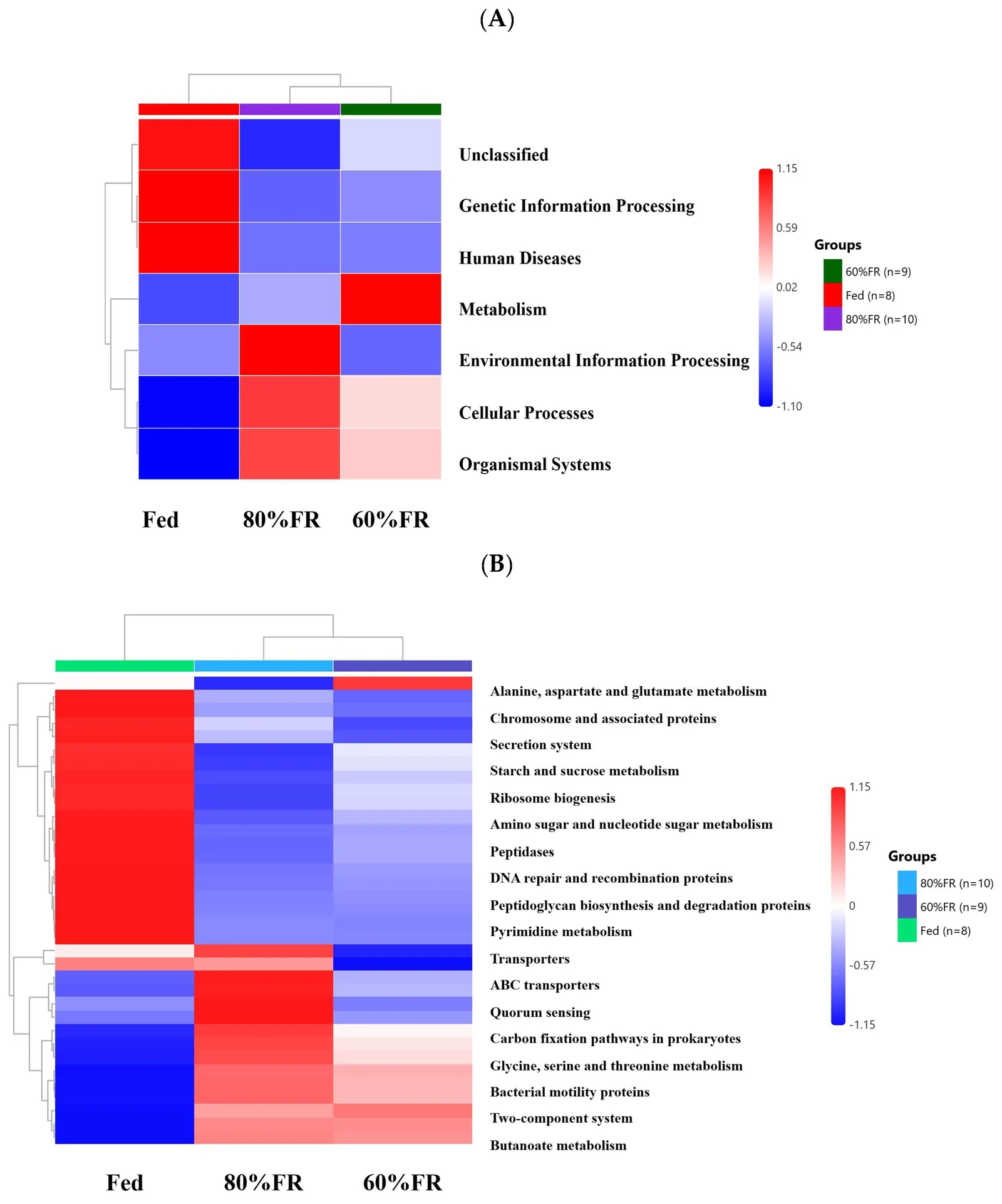
Clustering heatmap of relative abundance of Tax4Fun functional annotations based on level 1 (**A**) and level 3 (**B**) in Brandt’s voles.

At the phylum levels, the abundance of Firmicutes was positively correlated with MID and PLT, Actinobacteriota was positively correlated with MID, and Campylobacterota positively with Thymus. However, the abundance of Bacteroidota was negatively correlated with MID and IFN-γ, Euryarchaeota was also negatively correlated with RBC and total fat mass, and Campylobacterota negatively with HGB (Figure 15).

**Figure 16:**
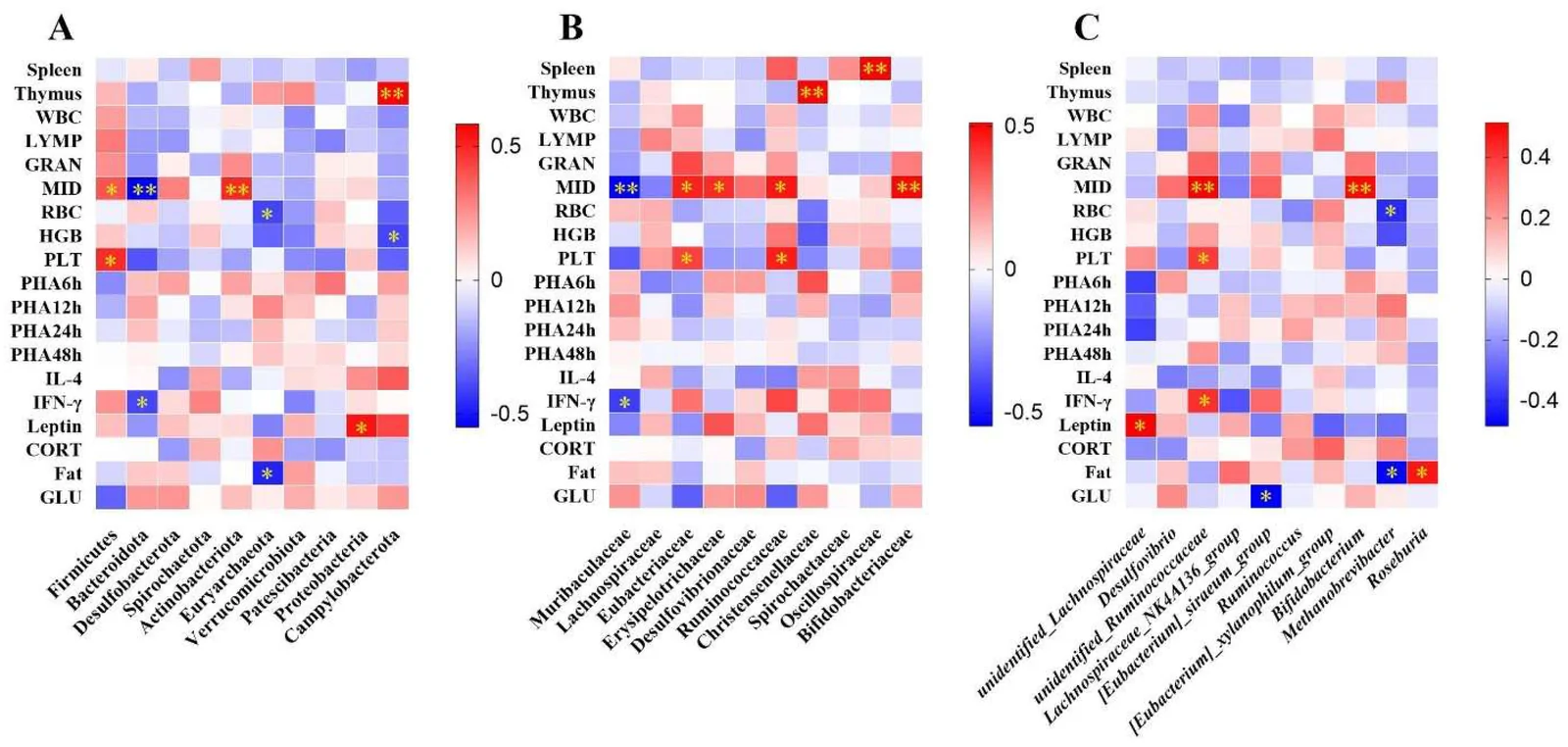
Correlation heatmap between gut microbiota and immune indicators at phylum level (**A**), family level (**B**) and genus level (**C**) in Brandt’s voles. One star (*) indicates *p* ˂ 0.05, and two stars (**) indicate *p* ˂ 0.01.

At the family level, spleen showed a positive correlation with Oscillospiraceae; thymus showed a positive correlation with Christensenellaceae; MID showed a positive correlation with Erysipelotrichaceae, Eubacteriaceae and Ruminococcaceae; and PLT showed a positive correlation with Eubacteriaceae and Ruminococcaceae. However, MID showed a negative correlation with Muribaculaceae, and IFN-γ showed a negative correlation with Muribaculaceae (Figure 16).

## 4. Discussion

In the present study, food restriction decreased body mass and total body fat mass in Brandt’s voles. Immunological parameters including the wet masses of the thymus and spleen, PHA response, and WBC and its related immune cells including lymphocytes, GRAN, MID and IL-4 level were not affected by food restriction, indicating moderate food shortage did not affect immune function in this species. Similar results were observed in other studies in which food restriction had no effect on cellular immunity in striped hamsters and deer mice [6,13]. However, other investigators found that cellular immunity was suppressed [8] or enhanced [5,39]. In addition, some researchers have reported that food restriction decreased WBC [6], or increased WBC [40], or had no effect on WBC [13]. The different results may be due to differences in the degree and duration of food restriction, and species used. The levels of blood glucose, leptin, and CORT were not impacted by food restriction, which might account for immune stability under moderate food shortage. Exceptionally, IFN-γ levels in the 80% FR group were the highest among the three groups. Different degrees of food restriction did not affect the α-diversity of the gut microbiota, indicating they had no effect on the diversity or richness of the gut microbiota. However, β-diversity showed a divergence between Fed group and FR groups, suggesting that food restriction influenced the structure and composition of the gut microbiota.

### 4.1. Effect of Food Restriction on Body Mass

With the time of food restriction increased, body mass gradually decreased, and the greater the degree of food shortage, the greater the reduction in body mass. This is common across many species such as striped hamsters [13] and *Eothenomys miletus* [41] and in other research in this species [30]. Animals usually mobilize their energy reserves (i.e., body fat) to adapt to food shortage. Although body compositions including wet carcass mass and total body fat mass did not differ statistically among the three groups, total body fat mass in the 80% and 60% FR groups decreased by 7.7% and 23.7%, respectively, in contrast with the Fed group. In a few species, body mass could remain stable under food shortage, such as *Acomys russatus* [42]. It seems that different species adopt distinct strategies in response to food shortage. The wet masses of most visceral organs in voles were unaffected by food restriction, which is similar to striped hamsters [13], but different to red-backed voles and *Eothenomys miletus* [43], rats and *Pycnonotus sinensis* [44,45]. The discrepancies among these studies may be attributed to the differences in the experimental regime, species used, and the degree and duration of food restriction.

### 4.2. Energy Status and Immunity Under Food Restriction

Food restriction usually regulates immune function through bioenergetic pathways. Body energy status including fat mass and metabolic fuels such as blood glucose can provide energy for costly physiological processes such as immune responses [46,47,48]. In addition, white adipose tissues are also endocrine and immune organs [24,49]. Animals having low energy storage might allocate less energy to immune responses than those with higher energy storage [50]. Consequently, a decrease in fat weight may suppress immune function [38,51]. In the present study, food restriction did not affect subcutaneous, mesenteric, retroperitoneal, perigonadal or total body fat masses and their respective percentage contents in Brandt’s voles, which was similar to striped hamsters under food restriction [13] but not consistent with other research in which food restriction reduced fat content [52,53]. Moreover, glucose is an important metabolic fuel which provides energy for maintaining and activating the immune system [54]. In our study, blood glucose levels remained stable in Brandt’s voles under food restriction. The positive correlation between blood glucose and PHA response after 12 h of PHA injection suggests that changes in cellular immunity are closely related to the levels of blood glucose. Moreover, both total body fat mass and blood glucose levels were not correlated with thymus and spleen, the majority of PHA responses, and WBC and its related immune cells including lymphocytes, GRAN, MID and IL-4 level, implying that stable body energy status may account for immune function being unaffected by food shortage in this species.

### 4.3. Hormone Profiles and Immunity Under Food Restriction

Both leptin and corticosterone are crucial hormones which regulate immune function. Leptin often has an enhancing effect on immune responses [23,55,56], and hence lower leptin concentration could reduce immune responses [57,58]. Leptin level was not influenced by food restriction in voles, and it was not correlated with most immunological parameters, implying stable leptin levels might be an important reason for immune function being unaffected by food shortage in Brandt’s voles.

Corticosterone usually increases under stressful conditions such as food shortage [6,7,59]. CORT often has a suppressive impact on immune responses [25,60,61]. CORT levels were not affected by food restriction, being inconsistent with other studies in which corticosterone levels increased under food restriction [6,7,59]. Other investigators have found that food restriction reduced the concentration of cortisol [5] or corticosterone [1]. No correlations were observed between corticosterone levels and immune organs, cellular immunity, and WBC-related cells including LYMP, GRAN and MID. Consequently, the lack of change in corticosterone concentration may account for the stable immunity under food restriction in Brandt’s voles.

### 4.4. Gut Microbiota and Immunity Under Food Restriction

The Chao1 index is used to assess community richness, and its value is higher, indicating greater species richness [29]. The Simpson index is indicative of community diversity and evenness, and the Shannon index is also used to evaluate the total number of taxa and their relative proportions within the community sample [43]. In the present study, food restriction had no influence on the α-diversity indices Chao1, Shannon, Pielou_e and Simpson, indicating the microbial community diversity and richness of the gut microbiota in the Brandt’s voles. This result was not consistent with other studies, in which a 50% food-restricted diet for 21 days in humans led to increased richness and diversity of the gut microbiota [62], a 21-day 80% food restriction decreased microbial richness in striped hamsters [63], and food restriction increased the α-diversity of the gut microbiota in mice [64].

Firmicutes and Bacteroidota are the dominant phyla in Brandt’s voles. The former primarily break down structural carbohydrates, while the latter mainly degrade non-structural carbohydrates and proteins [65]. Both Firmicutes and Bacteroidota play an active role in regulating animal metabolism and modulating the immune system through the production of short-chain fatty acids (SCFAs). At the family level, Muribaculaceae and Lachnospiraceae are the dominant families. The former mainly produce SCFAs from both endogenous and exogenous host polysaccharides, whereas the latter constitute a core component of the gut butyrate-producing microbiota and are associated with the energy supply to colonic epithelial cells, playing a crucial role in maintaining the integrity of the intestinal barrier [66]. In the present study, food restriction did not impact the relative abundance of all phyla including Firmicutes and Bacteroidota, and most families and genera in voles in the present study. For example, the function of Firmicutes, the most abundant phylum, is to promote fiber degradation and improve energy utilization efficiency, and hence the decrease in its relative abundance is generally considered to inhibit the host’s ability to derive energy from the diet [65]. Some studies have reported an elevated Firmicutes/Bacteroidota ratio in obese individuals, which decreases under food restriction [67]. Exceptionally, food restriction significantly reduced the relative abundance of Erysipelotrichaceae in Brandt’s voles, which belongs to a bacterial family within the Firmicutes. This family mainly participates in the degradation of complex polysaccharides and the production of short-chain fatty acids (SCFAs) in the gut, which plays a crucial role in host metabolic regulation and inflammatory diseases. The decrease in Erysipelotrichaceae in the food restricted group implied a reduced ability to obtain energy in voles. Some researchers have confirmed that the increase in the abundance of Erysipelotrichaceae is associated with diet-induced obesity [3,68]. Immunological indicators, including thymus and spleen weights, cellular immune levels, and IL-4 and IFN-γ, showed no correlations at the phylum, family or genus levels, suggesting that the stability of the gut microbiota under food shortage conditions may be one of the reasons why the immune function of the Brandt’s voles is unaffected by food restriction. In general, these results showed Brandt’s voles could maintain stable gut microbiota under moderate food shortage.

Beta diversity analysis showed a divergence between the Fed and FR groups, suggesting that food restriction altered the structure and composition of the gut microbiota. This finding was compatible with another study in which food restriction had an impact on the gut microbiota structure in striped hamsters [63]. LEfSe analysis and functional prediction indicate that food restriction increased the abundance of the order Eubacteriales and the family Eubacteriaceae in the 80% FR group compared with the Fed and 60% FR groups. The order Eubacteriales and its subordinate families are capable of producing short-chain fatty acids (SCFAs), particularly butyrate and acetate, which play a crucial role in maintaining intestinal barrier function and regulating immunity [26]. Tax4Fun functional annotation relative abundance clustering analysis showed that some pathways exhibited differences between the FR and Fed groups, whereas they were similar between the 80% FR and 60% FR groups. For instance, in contrast with the Fed group, 80% FR upregulated environmental information processing, cellular processes and organismal systems, and 60% FR enhanced metabolic function at level 1. These results suggested that food restriction might alter the functional profiles in gut microbiota. The 60% FR group increased the overall relative abundance of predictive pathways associated with metabolism, and 80% FR also significantly enhanced the overall relative abundance of predictive pathways associated with ABC transporters, carbon fixation, glycine, serine and threonine metabolism, two-component systems, and butyrate metabolism. These changes in the functional pathways suggested variation in host physiological processes, which may be induced by gut microbiota in response to food restriction. Their exact relationship remains unclear and requires further investigation in future.

### 4.5. Conclusions

In summary, food restriction decreased body mass and total body fat mass in Brandt’s voles. Immunological parameters including the wet masses of the thymus and spleen, PHA response, and WBC and its related immune cells including lymphocytes, GRAN, MID and IL-4 level were not affected by food restriction, implying voles could maintain stable immune responses under moderate food shortage. Energy status including body fat mass and blood glucose, and hormones such as leptin and CORT were not influenced by food restriction and they were almost not correlated with all immunological parameters. These results suggested that stable energy status and hormone levels might account for immune stability under different degrees of food shortage. Microbial community diversity and richness of the gut microbiota were also not affected, and the relative abundance at most phylum, family and genus levels also remains stable, which may be another reason for immune stability in Brandt’s voles under food shortage. Exceptionally, IFN-γ level in the 80% FR group was the highest among the three groups. Different degrees of food restriction did not affect the α-diversity of the gut microbiota, indicating they had no effect on the diversity or richness of the gut microbiota. However, β-diversity showed a divergence between Fed group and FR groups, suggesting that food restriction influenced the structure and composition of the gut microbiota.

## Figures and Tables

**Table 1:** Effect of different degrees of food restriction on the body composition in Brandt’s voles.

Parameters	Fed Group	80% FR Group	60% FR Group	Statistical Summary
Sample size	8	10	9	*p*
Initial mass (g)	55.5 ± 2.9	53.8 ± 2.0	55.1 ± 2.4	0.866
Final mass (g)	56.7 ± 3.7	53.3 ± 2.5	51.6 ± 2.9	0.515
Wet carcass mass (g)	40.5 ± 2.7	37.9 ± 1.8	35.7 ± 2.4	0.376
Subcutaneous fat (g)	1.17 ± 0.22	1.00 ± 0.17	0.83 ± 0.18	0.452
Subcutaneous fat content (%)	2.00 ± 0.27	1.81 ± 0.25	1.51 ± 0.27	0.448
Mesenteric fat (g)	0.22 ± 0.03	0.26 ± 0.03	0.21 ± 0.02	0.484
Mesenteric fat content (%)	0.39 ± 0.03	0.47 ± 0.04	0.41 ± 0.02	0.199
Retroperitoneal fat (g)	0.49 ± 0.11	0.47 ± 0.11	0.36 ± 0.07	0.573
Retroperitoneal fat content (%)	0.83 ± 0.15	0.85 ± 0.15	0.67 ± 0.12	0.611
Perigonadal fat (g)	0.85 ± 0.15	0.79 ± 0.11	0.69 ± 0.11	0.667
Perigonadal fat content (%)	1.46 ± 0.17	1.47 ± 0.16	1.30 ± 0.17	0.720
Total body fat (g)	2.74 ± 0.48	2.53 ± 0.38	2.09 ± 0.36	0.532
Total body fat content (%)	4.68 ± 0.55	4.60 ± 0.51	3.90 ± 0.52	0.528

Data are means ± SE. FR indicates food restriction.

**Table 2:** Effect of different degrees of food restriction on wet organ masses in Brandt’s voles.

Parameters	Fed Group	80% FR Group	60% FR Group	Statistical Summary
Sample size	8	10	9	*p*
Brain (g)	0.576 ± 0.018 ^a^	0.507 ± 0.016 ^b^	0.595 ± 0.018 ^a^	0.004
BAT (g)	0.333 ± 0.045	0.362 ± 0.04	0.3 ± 0.045	0.602
Heart (g)	0.304 ± 0.017	0.296 ± 0.015	0.254 ± 0.017	0.094
Lungs (g)	0.472 ± 0.056	0.426 ± 0.05	0.414 ± 0.056	0.736
Thymus (g)	0.013 ± 0.005	0.01 ± 0.005	0.007 ± 0.005	0.740
Liver (g)	2.318 ± 0.156	2.391 ± 0.14	2.042 ± 0.156	0.250
Spleen (g)	0.053 ± 0.008	0.049 ± 0.007	0.044 ± 0.007	0.720
Kidney (g)	0.559 ± 0.028	0.534 ± 0.025	0.538 ± 0.028	0.786
Adrenal gland (g)	0.019 ± 0.003	0.023 ± 0.003	0.02 ± 0.003	0.663
Stomach with its content (g)	0.641 ± 0.134	0.748 ± 0.12	0.544 ± 0.134	0.536
Small intestine with its content (g)	1.579 ± 0.142	1.446 ± 0.127	1.55 ± 0.142	0.763
Length of small intestine (cm)	26.8 ± 0.8	28.9 ± 0.8	28.1 ± 0.8	0.210
Cecum with its content (g)	2.926 ± 0.587	2.867 ± 0.527	4.211 ± 0.587	0.198
Length of cecum (cm)	7.8 ± 0.7	9.3 ± 0.6	9.2 ± 0.7	0.270
Colon (g)	0.753 ± 0.133	0.743 ± 0.119	1.012 ± 0.133	0.275
Length of colon (cm)	20.3 ± 1.05	20 ± 0.94	21.5 ± 1	0.533
Testis (g)	1.049 ± 0.041	1.039 ± 0.037	1.068 ± 0.041	0.874
Epididymis (g)	0.216 ± 0.02	0.185 ± 0.018	0.169 ± 0.02	0.258
Seminal vesicle (g)	0.791 ± 0.084	0.753 ± 0.075	0.595 ± 0.084	0.230

Data are means ± SE. Values for a specific parameter that shares different superscripts are significantly different at *p* < 0.05, determined by a GLM with initial body mass as the covariate and Bonferroni post hoc tests. Different letters (i.e., a or b) in the same line indicate significant differences between the groups. FR indicates food restriction.

**Table 3:** Correlation analysis between energy status and immune indicators in Brandt’s voles.

	Spleen	Thymus	WBC	LYMP	GRAN	MID	RBC	HGB	PLT	PHA6 h	PHA12 h	PHA24 h	PHA48 h	IL-4	IFN-γ	Leptin	CORT	Fat	Glc
Spleen	1																		
Thymus	−0.15	1																	
WBC	−0.11	−0.039	1																
LYMP	−0.10	0.055	0.931 **	1															
GRAN	−0.04	−0.163	0.175	0.045	1														
MID	0.08	−0.217	0.199	0.031	0.480 *	1													
RBC	0.33	−0.255	0.566 **	0.460 *	−0.039	−0.006	1												
HGB	0.34	−0.318	0.622 **	0.488 **	0.044	0.236	0.914 **	1											
PLT	0.38 *	−0.066	0.456 *	0.488 **	0.113	0.355	0.433 *	0.563 **	1										
PHA6 h	−0.11	0.423 *	0.065	−0.111	0.01	0.156	0.198	0.165	−0.19	1									
PHA12 h	−0.23	0.447 *	0.069	0.034	−0.046	−0.137	0.137	0.011	−0.28	0.688 **	1								
PHA24 h	−0.08	0.099	−0.055	−0.06	−0.016	−0.119	0.061	0.113	−0.26	0.374	0.564 **	1							
PHA48 h	−0.04	0.02	0.111	0.041	−0.155	0.025	0.183	0.202	−0.08	0.342	0.274	0.624 **	1						
IL-4	−0.20	0.485 *	0.263	0.169	0.223	−0.151	0.046	0.005	−0.21	0.436 *	0.266	−0.019	0.002	1					
IFN-γ	−0.0099	0.003	0.221	0.22	−0.095	0.382 *	0.037	0.105	0.32	0.018	−0.052	−0.158	0.101	−0.15	1				
Leptin	0.15	0.188	−0.157	−0.179	−0.184	0.209	−0.095	−0.011	0.03	0.083	−0.141	−0.174	0.009	0.29	−0.09	1			
CORT	−0.01	−0.282	0.108	0.144	−0.082	0.054	−0.054	−0.055	−0.12	−0.045	0.167	0.04	0.052	0.007	0.24	−0.13	1		
Fat	0.03	−0.109	−0.03	0.038	0.183	−0.226	0.117	0.074	−0.07	−0.241	−0.033	0.115	−0.316	−0.06	−0.25	−0.21	−0.30	1	
Glc	−0.15	0.134	0.035	0.033	−0.163	−0.164	0.127	−0.04	−0.15	0.328	0.481 *	0.124	0.301	0.22	0.15	0.14	0.26	−0.002	1

Data are correlation coefficients. One star (*) indicates *p* < 0.05, and two stars (**) indicate *p* < 0.01. Note: WBC: white blood cells; LYMP: lymphocytes; GRAN: neutrophil granulocytes; MID: intermediate cells; RBC: red blood cells; HGB: hemoglobin concentration; PLT: platelets; PHA6 h, 12 h, 24 h, and 48 h indicate PHA responses 6 h, 12 h, 24 h and 48 h after injection of PHA solution, respectively; IFN-γ: interferon γ; IL-4: interleukin-4; CORT: corticosterone; Fat: total body fat mass; Glc: blood glucose.

**Table 4:** Effect of different degree of food restriction on the hematological parameters in Brandt’s voles.

Parameters	Fed Group	80% FR Group	60% FR Group	Statistical Summary
Sample size	8	10	9	*p*
White blood cells (WBC) (10^9^/L)	5.35 ± 1.06	5.59 ± 0.90	4.94 ± 0.65	0.864
Lymphocytes (LYMP) (10^9^/L)	4.94 ± 1.05	4.98 ± 0.84	4.01 ± 0.85	0.692
Percentage of lymphocytes (LYMP%) (%)	92.3 ± 3.4	88.0 ± 3.1	79.2 ± 10.5	0.385
Neutrophile granulocytes (GRAN) (10^9^/L)	0.091 ± 0.041	0.124 ± 0.047	0.176 ± 0.110	0.729
Percentage of neutrophile granulocytes (GRAN%) (%)	1.66 ± 0.63	2.44 ± 0.60	3.83 ± 2.25	0.559
Intermediate cells (MID) (10^9^/L)	0.314 ± 0.176	0.485 ± 0.179	0.251 ± 0.083	0.529
Percentage of intermediate cells (MID%) (%)	6.01 ± 2.81	9.55 ± 2.68	5.88 ± 2.04	0.501
Red blood cells (RBC) (10^12^/L)	11.03 ± 0.87	9.94 ± 0.25	10.40 ± 0.70	0.482
Hemoglobin concentration (HGB) (g/L)	178.1 ± 15.7	165.6 ± 7.8	171.1 ± 11.4	0.750
Platelets (PLT) (10^9^/L)	433.9 ± 39.1	425.8 ± 29.9	402.2 ± 29.4	0.782

Data are means ± SE. FR indicates food restriction.

## Data Availability

The original contributions presented in this study are included in the article. Further inquiries can be directed to the corresponding author.
